# Does the Use of Crowdsourced Listeners Yield Different Speech Intelligibility Results Than In-Person Listeners for Typically Developing Children?

**DOI:** 10.1044/2025_JSLHR-25-00391

**Published:** 2026-01-29

**Authors:** Heather D. Salvo, Tristan J. Mahr, Carly Sandgren, Heather Mabie, Katherine C. Hustad

**Affiliations:** a Waisman Center, University of Wisconsin–Madison; b Department of Communication Sciences and Disorders, University of Wisconsin–Madison

## Abstract

**Purpose:**

We examined the performance of crowdsourced listeners compared with in-person listeners on the measurement of speech intelligibility for typically developing children. We used three different in-task quality check criteria to screen listeners and examined between-listener intelligibility differences and interrater reliability under each criterion. We also examined how crowdsourced intelligibility results compared with in-person results.

**Method:**

Sixty neurotypical children between ages 2;6 and 9;11 (years;months), drawn from Hustad et al. (2021), contributed speech samples. We used the online platform, Prolific, to collect intelligibility data from five crowdsourced listeners per child (*N* = 300 total) and compared scores with in-person results from two listeners per child. We used intraclass correlation coefficients (ICCs) and computed pairwise differences among listener groups for each of three in-task quality criteria groups and the in-person group. We modeled intelligibility as a function of listener source (in-person vs. crowdsourced) and child age using mixed-effects regression with smoothing splines.

**Results:**

Lower ICCs and larger between-listener differences were observed for crowdsourced compared to in-person listeners, regardless of in-task quality check criteria, but in-task quality check criteria reduced the disparity. Crowdsourced listeners produced intelligibility scores that were up to 7 percentage points lower than in-person listeners, even under the most stringent in-task quality check criterion. Results showed the same patterns of change with children's age as in-person listener findings. Children with midrange (65%–83%) intelligibilities were the most negatively impacted by the use of crowdsourced listeners.

**Conclusions:**

Rigorous in-task quality check criteria improved crowdsourced listener data. Speakers with midrange intelligibility were the most negatively impacted by the use of crowdsourced listeners, with an intelligibility difference of about 7 percentage points. Intelligibility data obtained with crowdsourced listeners should be interpreted with caution, and future studies should evaluate how crowdsourced intelligibility data differs from in-person data for disordered populations of speakers.

**Supplemental Material:**

https://doi.org/10.23641/asha.31101451

Crowdsourced listeners who participate in online research are widely used for the study of speech in both disordered and nondisordered populations. Studies have used crowdsourced listeners to examine a range of speech-related variables including speech intelligibility in dysarthria (Ziegler et al., 2021), listener training and adaptation in dysarthria (Lansford et al., 2016), overall speech quality (Cooke & García Lecumberri, 2021), articulation in children (McAllister Byun et al., 2015), perceptual features of dysarthric speech (McAllister et al., 2023), and features of cleft palate speech (Sescleifer et al., 2020), to name a few. Research suggests that results from crowdsourced listeners are generally consistent with findings from in-person listeners with regard to relative patterns of results and the effects of experimental manipulations (Lansford et al., 2016; Lechler & Wojcicki, 2024). However, some studies have also suggested that crowdsourced listeners may perform more poorly than in-person counterparts, owing to reduced experimental control and, thus, greater variability among listeners (Cooke & García Lecumberri, 2021; Lechler & Wojcicki, 2024; Yamamoto et al., 2021). Variability in study findings may be related, at least in part, to nuances pertaining to differences in research questions, research design, and measures/variables of interest; thus, research that addresses specific use cases for crowdsourced listeners is necessary.

In the present study, we compare intelligibility findings from a large-scale normative study of speech intelligibility development in typical children based on in-person listeners (Hustad et al., 2020, 2021) with intelligibility findings for a subset of the same children based on crowdsourced listeners. We also examine how different listener variables (i.e., criteria for data screening) and how different child variables (age, intelligibility) might impact findings. Our key motivation was to understand the extent to which intelligibility findings would be consistent and, therefore, generalizable across listener sources for the study of speech intelligibility in children and to quantify factors that might impact differences. Given the current widespread use of crowdsourcing for listening studies, this information provides a necessary foundation for considering the larger intelligibility literature that includes both in-person and crowdsourced intelligibility studies. Crowdsourced listeners are not yet used in clinical practice to obtain intelligibility information. However, this method may be on the horizon, providing another important context in which it is necessary to understand how intelligibility results from crowdsourced listeners compare to normative results from in-person listeners.

## Pros and Cons of Crowdsourced Listeners

Crowdsourcing offers many advantages in the study of speech. In particular, crowdsourcing methods cost considerably less than in-person methods in terms of participant payment and experimenter time. They are scalable in that large amounts of listener data can be obtained in a matter of hours or days, whereas in-person data collection can take weeks or months. Crowdsourced listener pools are broader and larger with regard to geographic reach, age, ethnicity, and other participant demographic variables as compared with in-person local participant pools. Ziegler et al. (2021) suggests that crowdsourced listeners provide high ecological validity in that they represent real-world unfamiliar listeners who are similar to everyday listeners that speakers may encounter.

Crowdsourced listeners also have some significant limitations. Critically, there is reduced experimental control across a range of variables (Harel et al., 2017; Ziegler et al., 2021). For example, the experimental setup can vary widely among listeners, including the quality of headphones or speakers, bandwidth of internet connections, fidelity of the speech signal, and noise and distraction in the listening environment. In addition, there may be technical challenges related to administration and completion of an experiment that render data unusable. Anonymity of listeners is also a limitation. Crowdsourcing relies on trust that listeners are accurately reporting their demographic information and are engaging in good faith efforts to complete tasks to the best of their ability. There is also a risk of *spamming*, which refers to crowd workers who are motivated to complete tasks quickly for financial gain and, thus, may provide random, repetitive, or stereotypical responses; disregard task instructions; and generally perform poorly on tasks in the interest of rapid completion (Ziegler et al., 2021). Bots, or automated responders, are another threat to the validity of crowdsourced results. Crowdsourced data, therefore, are often noisy, with more variability than in-person data, leading, in some cases, to lower absolute intelligibility scores relative to in-person laboratory data (Lechler & Wojcicki, 2024).

## Maximizing the Quality of Crowdsourced Listener Data

Across the literature, several approaches have been used to maximize the quality of crowdsourced data. Here, we consider prescreening approaches, in-task quality checks, performance-based criteria, and post-task data filtering.

Prescreening approaches have been used to narrow the participant pool prior to engaging with experimental tasks. The primary goal of prescreening is to recruit individuals who are likely to provide reliable and valid responses. Methods include IP address restrictions to particular geographic locations; the use of self-reported language background questions to control the desired native language of participants; the use of self-reported hearing status questions; validations of equipment usage, including headphone checks; and self-report of headphone use (Lechler & Wojcicki, 2024; McAllister Byun et al., 2015; Sescleifer et al., 2018). Furthermore, some platforms allow selection of crowd workers based on approval ratings from prior tasks; Lansford et al. (2016) obtained intelligibility data that were consistent with in-person speech findings by requiring crowd workers to have “master” designation on the Amazon Mechanical Turk platform. Note, however, that other studies have not found the “master” designation to be necessary for ensuring quality control in crowdsourced research tasks (Loepp & Kelly, 2020). In-task quality checks are activities embedded directly within experimental tasks to monitor participant attentiveness and adherence to instructions while they are completing the experiment (McAllister Byun et al., 2015; Newman et al., 2021; Peer et al., 2017; Sescleifer et al., 2018). In-task quality checks, also known as attention checks or catch trials, involve inserting specific items with predetermined, unambiguous answers into the task. These trials help identify inattentive participants by assessing their ability to recognize clearly correct or incorrect responses. Several studies have employed in-task attention checks (Fernández et al., 2019; Lechler & Wojcicki, 2024; Sescleifer et al., 2018). Researchers have used performance on in-task checks as criterion for exclusion. For example, McAllister Byun et al. (2015) used 20 catch trials per block and discarded data from participants who scored below chance on these trials. Similarly, Fernández et al. (2019) excluded participants who did not exceed chance-level performance on attentional catch trials, and Lechler and Wojcicki (2024) included data only from listeners who solved more than 80% of the validation questions correctly.

Performance-based criteria assess a participant's direct engagement with the experimental task. Unlike attention checks, which use separate, unambiguous items, performance-based checks evaluate whether participants' responses align with expected patterns or established benchmarks. McAllister Byun et al. (2015) employed an acoustic eligibility criterion in their study on /r/ perception. This criterion required participants' judgments to reflect known acoustic differences between correct and incorrect /r/ productions. If responses failed to meet this criterion, data were deemed unreliable and excluded. This approach ensures that participants not only pay attention but also demonstrate a level of perceptual discrimination necessary for valid results. Incentive-based validation questions have also been used as a type of performance-based quality control. Lechler and Wojcicki (2024) described a system where participants were rewarded with bonuses for correctly answering validation questions embedded within the task.

Post-task filtering involves reviewing and cleaning the data after it has been collected to remove any unreliable or low-quality responses. Ziegler et al. (2021) used a rigorous process of manual transcript review where all listener data were scrutinized for suspicious responses that could be indicative of inattentive or spamming behavior. When such cases were identified, data were removed, and new listeners were recruited. Suspected spammers were also restricted from future participation. Similarly, Lansford et al. (2016) eliminated listeners who did not provide a response for more than 20% of their items.

Collectively, studies using crowdsourced listeners have employed a range of strategies to reduce variability and increase fidelity and reliability of data from listeners. These studies provide a road map for an examination of crowdsourced listeners that incorporates pre-task screening, in-task quality checks, and post-task filtering methods to optimize listener data that can then be compared against data from in-person listeners.

In the present study, we examined child speech intelligibility findings, comparing crowdsourced intelligibility results to in-person intelligibility results on the same children. We examined the impact of three sets of in-task quality check criteria on resultant intelligibility data for each child and characterized differences between findings for each criteria level. We then examined how results obtained with the most stringent in-task quality check criteria compared with findings from in-person listeners. Research questions were as follows:

1. How do intelligibility results from crowdsourced listeners differ when three in-task quality criteria are employed (e.g., no criteria, 80% accuracy on in-task quality checks, and 90% accuracy on in-task quality checks)?
  - What are the average differences between listeners in each criterion?
  - What is the interrater reliability of in-person listeners and crowdsourced listeners for each criterion?
2. How do intelligibility results obtained from crowdsourced listeners under the most rigorous in-task quality criterion differ from results obtained from laboratory-based listeners?
  - To what extent are differences between crowdsourced and laboratory-based listeners impacted by child age and by child intelligibility?
  - How do children's age percentile ranks for intelligibility differ for crowdsourced versus laboratory-based listeners?

For comparisons within crowdsourced participants, we hypothesized that different in-task quality criteria would impact results such that more stringent screening criteria would lead to outcomes that most closely resembled in-person data. Specifically, we anticipated stronger interrater reliability among listeners who met higher in-task quality criteria (e.g., 90%) compared to those who met lower in-task quality criteria (e.g., 80% or no criteria). We expected that laboratory-based in-person listeners would provide intelligibility scores that were higher than those from crowdsourced listeners, but that this difference would be reduced when the most stringent in-task quality criteria were met. We expected the differences between in-person and crowdsourced listeners to be smaller for highly intelligible children (including older children) and for highly unintelligible children (including the youngest children), because ceiling and floor effects would reduce variability between listener types. We expected that the greatest differences between in-person and crowdsourced listeners would occur at the midpoint of the intelligibility continuum (and also the midpoint of the age continuum), where there is more ambiguity in the signal.

## Method

This study was approved by the institutional review board at the University of Wisconsin–Madison (No. 2020–1620). Informed consent was obtained from or on behalf of all participants.

### Participants

#### Typically Developing Children

This study included a subset of 60 children from a larger study of speech intelligibility development (*n* = 538); see Hustad et al. (2020, 2021). Because the population and recruitment methods are described in these earlier studies, we provide a cursory summary here. For the larger study, children were recruited via public postings, online advertisements, and a research registry at the University of Wisconsin–Madison. Inclusion criteria were (a) American English as the primary language in the home, (b) hearing within normal limits, and (c) speech and language within normal limits as indicated by formal screening. Children receiving intervention services for developmental or educational delays, and those with medical diagnoses related to development, were excluded.

**Figure 1.**
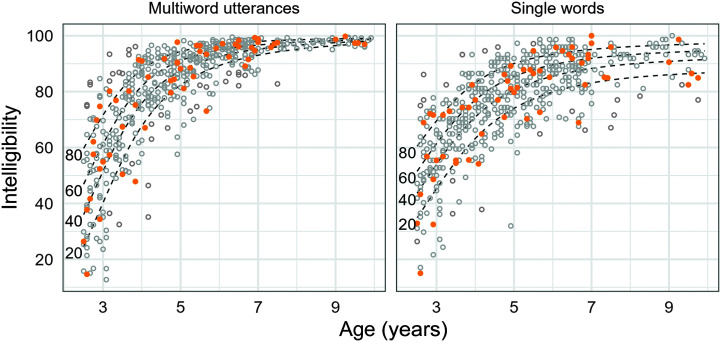
Intelligibility distributions for children included in the present study (orange points) relative to children in the larger study by Hustad et al. (2021) (gray points). Dashed lines represent the 20th, 40th, 60th, and 80th percentiles estimated from the larger sample.

Children in the sample reported in the current study ranged in age from 2;6 to 9;09 (years;months) and included 30 boys and 30 girls. Specifically, one boy and one girl were selected from each age band (2;6–2;11, 3;0–3;11, 4;0–4;11, 5;0–5;11, 6;1–6;11, 7;0–9;9) for the following intelligibility quintiles: 0–20th percentile, 21–40th percentile, 41–60th percentile, 61–80th percentile, and 81–100th percentile based on data from Hustad et al. (2021). This method allowed us to obtain a representative sample of children based on performance bands within age levels and sex. Table 1 shows the age and sex distribution of children included in the present study. Figure 1 shows the intelligibility distributions of children included in the present study relative to the larger normative sample based on in-person listeners for single-word and multiword intelligibility.

**Table 1.** Age and sex distribution of typically developing child speakers (*N* = 60).

Age group (years;months)	*n*	Boys	Girls	Age *M*	Age *SD* (months)
2;6–2;11	10	5	5	2;9	2
3;0–3;11	10	5	5	3;6	4
4;0–4;11	10	5	5	4;7	4
5;0–5;11	10	5	5	5;5	3
6;0–6;11	10	5	5	6;7	3
7;0–9;11	10	5	5	8;4	14

*Note.* Every age group included one boy and one girl from each of the following speech intelligibility percentile ranges: 0–20, 21–40, 41–60, 61–80, and 81–100.

#### Adult Listeners

Two groups of adult listeners made orthographic transcriptions of children's speech. One group made orthographic transcriptions in person in our laboratory at the University of Wisconsin–Madison; the other group made orthographic transcriptions via an online crowdsourcing platform.

For in-person listeners, we used orthographic transcription data published previously by Hustad et al. (2021) for the specific children included in the present study. As detailed in our previous papers, listeners were recruited from the University of Wisconsin–Madison student population through public postings. Listeners were required to (a) pass a pure-tone hearing screening at 20 dB for 250, 500, 1000, 4000, and 8000 Hz; (b) be between 18 and 45 years of age; (c) have no more than incidental experience listening to or communicating with persons having communication disorders; (d) be monolingual native speakers of American English; and (e) have no identified language, learning, or cognitive disabilities per self-report. Two different listeners heard each child for a total of 120 adults. Each listener heard speech samples from just one child during the task. The mean age of listeners was 20.8 (*SD* = 2.9) years.

For crowdsourced listeners, we used the platform Prolific (2024) for recruitment. Prolific is widely used for obtaining research participants (Douglas et al., 2023; Peer et al., 2017; Stipancic et al., 2025; Tripp et al., 2025). For this study, listeners were required to (a) report normal hearing, (b) be between 18 and 45 years of age, (c) be monolingual native speakers of American English, and (d) reside within the United States. On Prolific, we set filters for exclusion based on the country of residence, hearing difficulties, age, and language. We also excluded participants who had engaged with other studies from our lab. Listeners were only allowed to participate in this study one time, and only listened to one child. Because we expected that there would be more variability among crowdsourced listeners, we sampled a larger number of crowdsourced listeners than in-person listeners. We sought to collect data from five listeners per child, for a total of 300 listeners. However, following our research questions, which addressed the effects of three different sets of in-task quality check criteria on intelligibility results, it was necessary to collect data from 363 online crowdsourced listeners (191 males, 162 females, and 10 other or unspecified). Table 2 provides a summary of listener inclusion/exclusion for each of the three in-task quality criteria (no criterion, 80% accuracy on in-task quality checks, and 90% accuracy on in-task quality checks). For each in-task quality criterion group, the number of listeners that were considered strict exclusions (removed on the basis of failing to meet inclusion criteria, not completing the experiment, or experiencing a technological problem in their experiment) was about 10% of the target sample size. In-task quality data screening exclusions led to loss of an additional 5% of the target sample at the 80% criteria, and an additional 10% of the target sample at the 90% criteria. Table 3 provides demographic information for each group of listeners examined in this study.

**Table 2.** Crowdsourced listener totals by in-task quality check threshold.

In-task quality check criteria	Total listeners	Strict exclusions	Data screening exclusions	Included listeners
None	327	27	0	300
80%	(+15) 342	(+2) 29	(+13) 13	300
90%	(+21) 363	(+4) 33	(+17) 30	300

**Table 3.** Demographics of in-person and crowdsourced listeners by participation modality and data fidelity criteria standards.

Demographics		In person		Crowdsourced No screening		Crowdsourced in-task quality ≥ 80%		Crowdsourced in-task quality ≥ 90%	
Age	Range (years)	18–34		18–45		18–45		18–45	
	*M* (*SD*)	20.73 (2.9)		32.9 (7.0)		33.1 (6.9)		33.2 (7.0)	
		*n*	%	*n*	%	*n*	%	*n*	%
Sex	Male	31	26%	160	53.3%	158	52.7%	155	51.7%
	Female	89	74%	138	46%	139	46.3%	143	47.7%
	Did not provide	0	0%	2	0.7%	3	1%	2	0.7%
Race	Asian	14	12%	16	5.3%	18	6%	19	6.3%
	Black or African American	3	2.5%	55	18.3%	48	16%	45	15%
	More than one race	4	3.3%	13	4.3%	14	4.7%	14	4.7%
	Native Hawaiian or Pacific Islander	0	0%	1	0.3%	1	0.3%	1	0.3%
	White	99	82.5%	215	71.7%	219	73%	221	73.7%
Ethnicity	Hispanic/Latino	2	1.7%	15	5%	17	5.7%	16	5.3%
	Non-Hispanic/Latino	118	98.3%	285	95%	283	94.3%	284	94.7%

*Note.* When we impose in-task quality check criteria, more listeners must be recruited to reach the target of 300 included listeners. “Strict exclusions” are listeners who participated but were discarded due to failing to meet inclusion criteria, not completing the experiment, or experiencing a technological problem in their experiment. “Data screening exclusions” are listeners who scored below specific criteria on in-task quality checks.

### Materials and Procedures

#### Speech Samples From Children

Speech samples from children were a standard set of stimuli elicited by a research speech-language pathologist in a sound-attenuating suite. We used stimuli from the Test of Children's Speech (TOCS+; Hodge & Daniels, 2007) comprising 37 single-word productions and 60 multiword sentences varying in length from two to seven words. Stimulus items are publicly available via the open-access supplemental material of Hustad et al. (2021; DOI: 10.23641/asha.16583426.v1). Using a corpus of stimuli that was the same across all children allowed us to compare listener orthographic transcriptions against known target responses.

Children's speech was recorded using a professional quality digital audio recorder (Marantz PMD570) at a 44.1-kHz sampling rate (16-bit quantization) and a condenser studio microphone (Audio-Technica AT4040) positioned 18 in. from the child's mouth. Recordings were monitored in real time and recording levels were adjusted on a mixer (Mackie 1202 VLZ) to obtain optimized recordings. Digital recordings of children's speech were prepared for playback to listeners by separating each utterance into its own file, removing extraneous noises preceding or following the production of each utterance, and peak amplitude normalizing each utterance.

#### Intelligibility Measurement

All samples for each child were presented to in-person listeners and to online crowdsourced listeners, who transcribed orthographically what they heard in a self-paced task. In-person listeners were seated in a quiet room with peak audio output levels calibrated to 75 dB SPL; crowdsourced listeners were asked to wear headphones and to complete the experiment in a quiet room. Although recent studies outline procedures for headphone checks in crowdsourced listening paradigms (Milne et al., 2021; Woods et al., 2017), listeners did not complete a headphone check in the present study. For all listeners, child productions were presented in random order, with all single words presented in one block and all multiword utterances presented in a separate block; block order was counterbalanced.

For each listener of each child, we computed intelligibility as the proportion of words correctly identified by a listener. Aggregated intelligibility scores for each child were computed by averaging the utterance-level intelligibility scores. For example, we computed multiword intelligibility by averaging the intelligibility scores of the multiword utterances for each listener. Single-word intelligibility was calculated by averaging the intelligibility scores of the single-word utterances for each listener.

#### Post-Task Data Screening

Following data collection from each listener, demographic information was reviewed via self-reported responses to our study questionnaire that queried our inclusion criteria (i.e., hearing status, age, native language, geographic location). Next, listeners' orthographic responses were reviewed by members of the research team. Spelling errors were corrected, punctuation and extra spaces were edited, and abbreviations were written out in full to ensure accurate processing by our automated scoring system. Technical and other issues were identified in post-task data screening on the basis of written responses such as “no audio played” or when the identical response was written two or more consecutive times. Listeners who did not complete all prompts by writing an orthographic response were also identified. Listeners who failed to meet inclusion criteria, experienced a technological problem in their experiment, or did not complete the experiment were eliminated from the analysis pool in post-task data screening as strict exclusions.

#### In-Task Quality Checks

To prevent risks to data quality associated with crowdsourced listeners, we included in-task quality checks in the experiment for crowdsourced listeners; we used performance on these checks as data screening exclusions. In-task quality check items were used to assess task engagement and effort and to mitigate against inclusion of data from unreliable participants. Online participants were not explicitly informed of the presence of in-task quality check items to prevent potential influence on task performance. In-task quality check items were four single-word stimuli and four 4-word sentence stimuli, which were interspersed randomly within the child productions of single-word and multiword stimuli. Listeners orthographically transcribed these items, just as they did the child productions. All in-task quality check items were produced by an adult female with mature speech production abilities. In-task quality check items were similar in content and length to the words and sentences produced by children in the study. As with the child-produced items, we computed the intelligibility of the adult-produced in-task quality check items by averaging the utterance-level intelligibilities across items. The average intelligibility of in-task quality check items was used to screen intelligibility data from online listeners at three different accuracy levels—no in-task quality criteria applied, 80% accuracy on in-task quality check items, and 90% accuracy on in-task quality check items.

#### Statistical Analyses

To address Question 1, we computed interrater reliability for each listener group and each in-task quality criteria group with the intraclass correlation coefficient (ICC). We used a one-way random-effects model—ICC(1)—and computed these values using the psych R package (Version 2.4.12; Revelle, 2024). We also computed pairwise differences among listener groups and each in-task quality criteria group for a given child. In the crowdsourced sample, there were five listeners per child, yielding 10 listener pairs per child and 600 overall pairwise differences. We report mean differences across the 10 listener pairs. For the in-person listeners, with two listeners per child, there were 60 pairs.

To address Question 2, we modeled intelligibility as a function of listener source (in-person vs. crowdsourced) and child age using mixed-effects regression with smoothing splines. The model included an overall effect of the listener source. Because there were repeated measures at the child level (multiple listeners per child), we used by-child varying effects for each listener source. That is, when estimating a child's intelligibility, the model first estimates the intelligibility of a statistically typical participant of a given age and listener source. These typical estimates are then adjusted using a child-specific overall effect (i.e., by-child random intercept) and a child-specific listener source effect (i.e., by-child random slope).

We included age in our models using smoothing splines (Wood, 2017). Under a generic spline approach, a time series is decomposed into local curves (called “basis functions”) that are weighted and summed together to follow a nonlinear trend of the data. The number of these local curves determines the flexibility of the nonlinear trend, and adding more local curves allows for the fitted trend to become more “wiggly” and sensitive to local fluctuations in the data. Thus, a smoothing spline approach estimates and applies a penalty that restricts the amount of “wiggliness” in the smooth (i.e., the fitted trend). Because there were two sets of intelligibility scores—in-person listeners and crowdsourced listeners—we included two smooths. The first smooth estimated a baseline for in-person listeners and the second smooth estimated the difference between in-person and crowdsourced listeners.

Single-word intelligibility and multiword intelligibility were analyzed separately using different model families (binomial distribution and beta distribution, respectively). These are two different approaches for handling probabilities and proportions (i.e., intelligibilities). The binomial family models the number of successes from a given number of trials (total number of single words correctly transcribed). For multiword utterances, where it is not safe to assume that each word is an independent trial, we used the beta distribution to directly model proportions.

We fit the models using a Bayesian framework with weakly informative priors. For point estimates and intervals, we report the median and 95% equal-tail interval (2.5 and 97.5 quantiles) from the posterior distribution. Analyses were conducted in the R programming language (Version 4.5.0; R Core Team, 2025). Models were fit using the Stan programming language (Version 2.36.0; Stan Development Team, 2024) via brms (Version 2.22.0; Bürkner, 2017) and cmdstanr (Version 0.9.0; Gabry et al., 2024) R packages. Supplemental Material S1 provides the complete syntax for the two models as well as the workflow for computing marginal means from each model.

## Results

### Question 1: Impacts of In-Task Quality Check Criteria on Intelligibility Data

To address the first research question, we compared three in-task quality check scenarios: (a) no screening (first five crowdsourced listeners with complete data), (b) 80% in-task quality criterion (first five listeners with at least 80% intelligibility on in-task quality check items), and (c) 90% in-task quality criterion (first five listeners with at least 90% intelligibility on in-task quality check items). Table 4 displays summary statistics for the listener groups and in-task quality criteria groups. Descriptive data suggest that means and standard deviations of the pairwise differences decreased as the in-task quality check criteria increased. The average range—the average of the minimum and maximum values taken from each child—also narrowed with stricter screening thresholds. The pairwise differences for single words tended to be larger than for multiword items, but this feature might reflect the binary nature of scoring for single words. That is, for a single words, a transcription of an item has either 0% or 100% intelligibility, but for multiword items, listeners can be partially correct.

**Table 4.** Summary statistics of pairwise differences between listeners.

Listener set	Mean difference between listeners (*SD*)	Median (IQR)	Range	Average range
Single-word intelligibility				
In person	5.6 (5.4)	2.9 (5.4)	0.0–24.3	—
Crowdsourced, no screening	11.4 (11.7)	7.7 (12.8)	0.0–63.9	1.1–23.5
Crowdsourced, 80% criteria	9.7 (9.4)	7.7 (10.2)	0.0–63.9	0.8–19.8
Crowdsourced, 90% criteria	8.8 (7.9)	7.7 (10.3)	0.0–43.6	0.6–17.9
Multiword intelligibility				
In person	3.2 (3.2)	2.0 (4.4)	0.0–12.4	—
Crowdsourced, no screening	9.6 (10.4)	5.4 (11.0)	0.0–54.9	1.0–20.0
Crowdsourced, 80% criteria	7.8 (8.5)	4.7 (9.0)	0.0–46.0	0.8–16.5
Crowdsources, 90% criteria	7.2 (7.8)	4.5 (8.3)	0.0–46.0	0.8–15.2

*Note.* We computed all pairwise differences in intelligibility values among the listeners of each speaker. The range provides the overall range (smallest and largest pairwise differences observed across all listeners), and the average range provides the average minimum and maximum of pairwise differences for each child. Em dashes indicate data not available. IQR = interquartile range.

As expected, the average pairwise differences for in-person listeners were smaller than their crowdsourced counterparts, around half the magnitude for multiword intelligibility (3.2 vs. 7.2 percentage points) and two-thirds the magnitude for single-word intelligibility (5.6 vs. 8.8 percentage points) for the most rigorous in-task screening criterion (90%). Pairwise differences were considerably larger for the no-screening condition. We note two limitations for this pairwise comparison: First, the pairs of in-person listeners were previously screened so that the difference in the overall percentage of words correctly transcribed in each pair was no larger than 10 percentage points. When differences between listeners greater than 10 occurred, we recruited an additional listener and used the two listeners that were closest together. However, these differences occurred infrequently, with only four of 60 children in this subsample requiring an additional listener. Second, there were more crowdsourced listeners per child and, hence, more opportunities to recruit a listener who would provide a discrepant or extreme intelligibility score with large pairwise differences.

Table 5 shows two sets of ICC scores: ICC(1) measures the reliability of a single intelligibility score—or more specifically, the expected correlation of two scores from two randomly selected listeners for the same individual—and ICC(1,*k*) measures the reliability of the average of *k* scores. Reliabilities for the in-person listeners were almost always higher than the crowdsourced listeners, supporting the use of an in-person, laboratory-controlled gold standard. With no data screening, the reliability of a single online score was relatively low, ICC(1)_single-word_ = .681, ICC(1)_multiword_ = .813, but the reliability increased as the 80% and 90% data screening criteria were applied, ICC(1)_single-word_ = .817, ICC(1)_multiword_ = .888 at the 90% threshold. This improvement in reliability confirms that the data screening criteria removed some unreliable listeners. The ICCs for the averages of five online listeners, ICC(1, 5)_single-word_ = .957, ICC(1, 5)_multiword_ = .975, at the 90% threshold also approached or equaled in-person reliability, ICC(1, 2)_single-word_ = .955, ICC(1, 2)_multiword_ = .988. We note, however, the distinction between precision (reliability) and accuracy, as highly reliable intelligibility scores may be systematically different from in-person intelligibility scores.

**Table 5.** Intraclass correlation coefficients (ICCs) by in-task quality check criteria.

Intelligibility type and listener set	Listeners	Single score ICC(1) [95% CI]	Average score ICC(1, *k*) [95% CI]
Single-word intelligibility			
In person	2	.914 [.860, .947]	.955 [.925, .973]
Online, no screening	5	.681 [.583, .772]	.914 [.875, .944]
Online, 80% threshold	5	.763 [.681, .835]	.941 [.914, .962]
Online, 90% threshold	5	.817 [.749, .875]	.957 [.937, .972]
Multiword intelligibility			
In person	2	.977 [.961, .986]	.988 [.980, .993]
Online, no screening	5	.813 [.744, .872]	.956 [.936, .971]
Online, 80% threshold	5	.869 [.818, .912]	.971 [.957, .981]
Online, 90% threshold	5	.888 [.842, .925]	.975 [.964, .984]

*Note.* CI = confidence interval.

### Question 2: In-Person Versus Crowd-Sourced Listener Intelligibility

**Figure 2.**
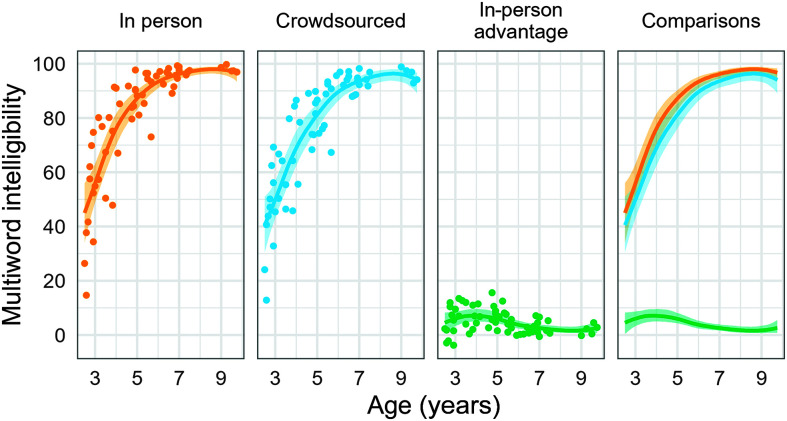
Expected multiword intelligibility for an average in-person listener, an average crowdsourced listener, and the difference between these averages, each as a function of age. Points represent the average intelligibility score (or difference in average scores) for a child. Note that crowdsourced listener data are for listeners who met the 90% in-task accuracy criterion.

**Figure 3.**
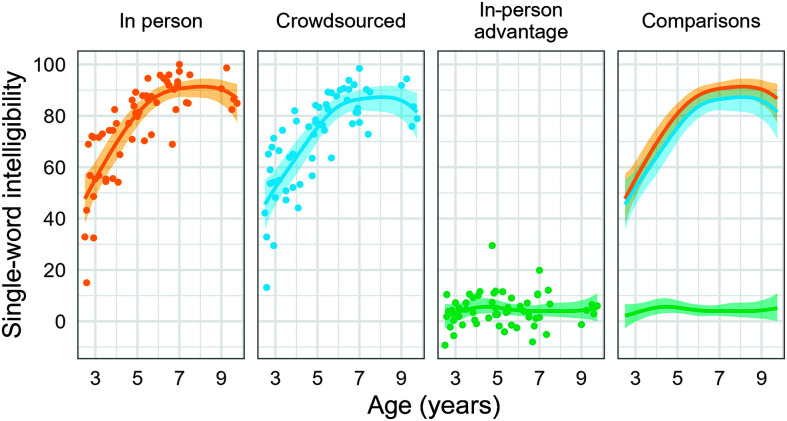
Expected single-word intelligibility for an average in-person listener, an average crowdsourced listener, and the difference between these averages. Points represent the average intelligibility score (or the difference in average scores) for a child. Note that crowdsourced listener data are for listeners who met the 90% in-task accuracy criterion.

Figures 2 and 3 show the expected intelligibilities for an average in-person listener, an average crowdsourced listener, and the difference between these averages, each as a function of age for listeners who met the 90% in-task intelligibility accuracy criterion. For multiword utterances, the difference in expected intelligibility was greatest at ages younger than 5;0, as shown in Figure 2 (panel 3). The maximum difference in expected intelligibility was 7.5 percentage points, 95% posterior interval [5.5, 10.0], and the estimated age at which the greatest difference emerged was 3;10 [2;7, 5;0]. After age 5;0, the expected difference between listener sources decreased, as intelligibility scores approached a ceiling of 100%. For example, the expected difference was 3.8 percentage points [2.4, 5.4] at age 6;0 and 1.8 percentage points [0.9, 3.2] at age 8;0.

For single words, the growth curve for the expected difference in intelligibility was a mostly flat line (see Figure 2, panel 3). That is, the difference between the two listener groups did not appear to be sensitive to the child's age. As a result, the age of maximum expected difference is highly uncertain, with a 95% posterior interval that effectively spans the age range of the sample [2;10, 9;9]. The maximum difference estimate, 6.9 percentage points [4.4, 11.3], however, was similar in magnitude to the value for connected speech.

**Figure 4.**
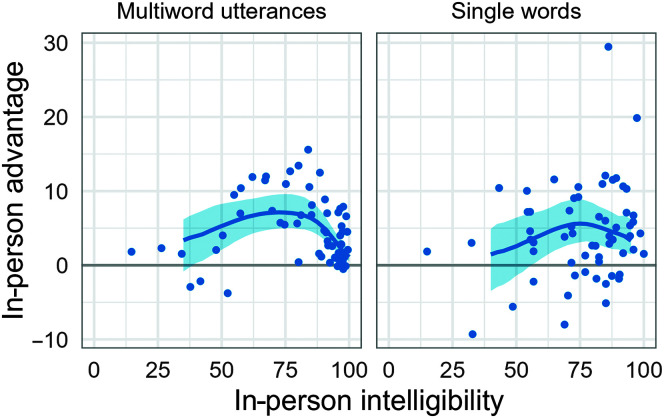
Relationship between in-person intelligibility and difference between in-person and crowdsourced intelligibility scores for listeners who met 90% in-task intelligibility accuracy criteria. The expected difference between listener types (in-person minus crowdsourced) is plotted as a function of the expected in-person intelligibility. The regression line and 95% posterior interval were derived from the growth curves in Figure 2 and Figure 3. Points represent observed differences, with one observation per child per panel.

Figure 4 visualizes in-person intelligibility versus the difference between in-person and crowdsourced intelligibility (i.e., the in-person advantage) for listeners who met the 90% in-task intelligibility accuracy criterion. To generate this figure, we computed a posterior distribution of growth curves as in Figures 2 and 3, but instead of aggregating by age, we binned the in-person intelligibility scores (rounded to the nearest 2.5 percentage points) and examined the in-person versus crowdsourced differences within each bin. This approach provided a set of plausible expected differences (in-person advantage scores) at each level of in-person intelligibility.

For multiword utterances, the expected difference was largest for in-person intelligibility levels between 65% and 80%. Specifically, the greatest difference occurred at 72.5% intelligibility, where in-person listeners had an advantage of 7.1 percentage points, 95% posterior interval [5.0, 9.5]. Similar expected differences occurred at 65.0% intelligibility (6.9, [4.5, 9.2]) and 80.0% intelligibility (6.9, [4.9, 9.4]). The expected difference decreased above 85% intelligibility, and for intelligibility levels of 95.0% and higher, the expected difference (and 95% posterior interval) fell below 5 percentage points.

For single-word utterances, the expected difference was largest for in-person intelligibility levels between 67.5% and 82.5%. The expected difference was greatest at 75.0% intelligibility, with an in-person advantage of 5.6 percentage points, 95% posterior interval [3.1, 8.9]. Compared to multiword utterances, there was a stronger floor effect. Below 55.0% intelligibility, there was not a statistically clear in-person listener advantage (that is, the 95% posterior interval included 0 as a plausible value for the difference). There was also not an apparent ceiling effect such that differences diminished at high intelligibilities. However, this lack of a ceiling effect was likely driven by a relatively small number of high-intelligibility observations, with only six children above 95% in-person intelligibility for single-word utterances compared to 20 children for multiword utterances.

**Figure 5.**
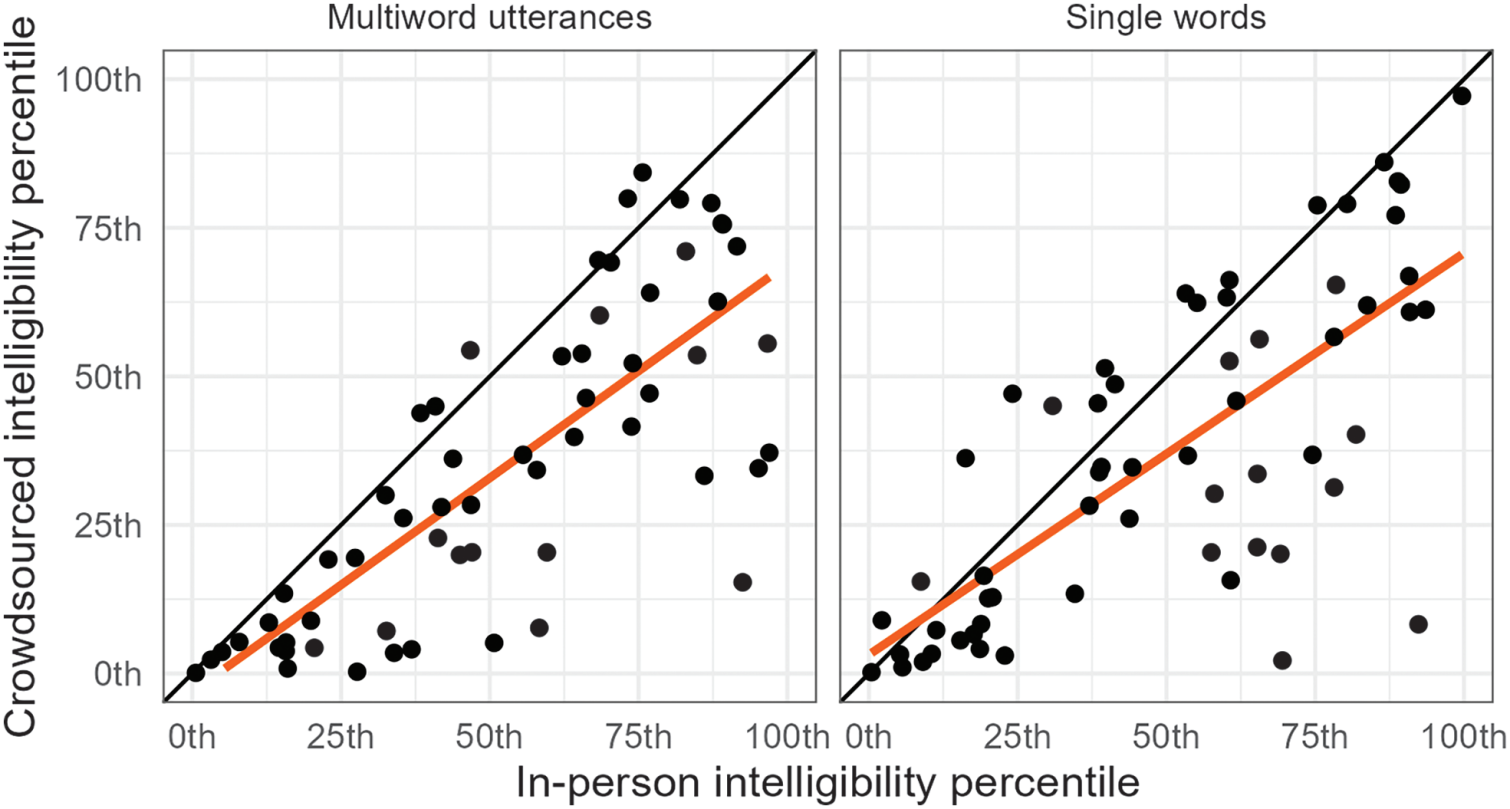
Comparison of age percentile ranks based on in-person intelligibility scores versus crowdsourced intelligibility scores. Intelligibility scores from each listener source were averaged together for each child. Crowdsourced listeners were the subset who met the 90% in-task accuracy criterion. The dark orange line is a regression line to illustrate the downward bias in crowdsourced intelligibility percentiles.

The above analyses examined the differences between in-person and crowdsourced intelligibility scores with respect to stimulus type (single-word and multiword utterances), speaker age, and average in-person intelligibility. As a final comparison, we examined the downstream effect of crowdsourced intelligibility scores on norm-referenced intelligibility assessment. That is, we compared intelligibility age percentiles computed using the average in-person intelligibility versus age percentiles computed from the averaged crowdsourced intelligibility. To do this, we used the growth curve percentile models for intelligibility established in Hustad et al. (2021) and plotted in Figure 1. Figure 5 shows age-based intelligibility percentile scores for individual children from the in-person listener data (*x*-axis) and the corresponding percentile scores based on crowdsourced listener data for the 90% in-task screening criterion (*y*-axis). Points falling below the black diagonal line indicate reduced percentile values for crowdsourced listeners. Results indicated that most children had decreased intelligibility age percentiles for connected speech (*n* = 54) and single words (*n* = 47) with crowdsourced listeners. On average, the relative decrease in percentile rank—calculated as (in-person − crowdsourced) / in-person—was 38.1% (*SD* = 30.9) for multiword utterances and 19.8% (*SD* = 61.5) for single words. Finally, we asked how these fluctuations in percentile rankings might affect clinical decision making. There were 13 participants who were above the 10th percentile for multiword intelligibility based on in-person scores but who were below the 10th percentile based on crowdsourced scores. There were nine analogous cases for single words. There was just one participant who made the opposite switch (from below the 10th percentile to above the 10th percentile).

## Discussion

In this study, we examined the performance of crowdsourced listeners compared with in-person listeners on the measurement of speech intelligibility for typically developing children. Specifically, we studied a representative sample of 60 neurotypical child speakers between the ages of 2;6 and 9;11 years;months, drawn from Hustad et al. (2021). We collected intelligibility data from five crowdsourced listeners per child (*n* = 300) and compared results with gold-standard in-person listener intelligibility data (*n* = 120) on the same children. We applied three different in-task quality check thresholds as a means to eliminate inattentive crowdsourced listeners and then examined how between-listener intelligibility differences and interrater reliability varied under each in-task quality check criterion. We then examined how crowdsourced intelligibility results under the most rigorous criteria compared with in-person results. This study revealed three main findings. (a) Laboratory-controlled in-person listener intelligibility data showed less variability and better reliability than crowdsourced intelligibility data on the same children regardless of in-task quality check criteria, but in-task quality check criteria reduced the disparity. (b) Crowdsourced listeners, on average, produced intelligibility scores that were up to 7 percentage points lower than in-person listeners, even when the most stringent in-task quality check criterion was applied, but results showed the same pattern of growth as in-person listener findings. (c) The difference between in-person and crowdsourced results was affected by the intelligibility of the particular child; speakers with midrange (65%–83%) intelligibilities were the most negatively impacted by use of crowdsourced listeners. These findings and their implications are discussed.

### In-Person Intelligibility Data Are Less Variable and More Reliable Than Crowdsourced Data

Listener variability is known to be an important issue in speech intelligibility research (Hustad et al., 2015). At the heart of listener variability is the question of generalizability; that is, to what extent is any one listener or group of listeners representative of other listeners that a speaker may encounter? A key goal both in research and in clinical practice is to provide an ecologically valid representation of a speaker's intelligibility that is robust to individual listener differences. For our in-person study on children's intelligibility development (Hustad et al., 2021), we used two listeners per child for efficiency, primarily due to the large sample of children (*n* = 538) and the onerous task of data collection from in-person listeners. To compensate for the small number of listeners per child, we examined mean differences between listeners of the same speaker. When there were between-listener differences of more than 10 percentage points, we obtained data from a third listener and used the two listeners that were closest together. For the subset of children included in the present study, this occurred only four times across 60 children, suggesting that listener performance was relatively homogenous, with mean differences between in-person listeners of 3.2 and 5.6 percentage points for multiword utterances and single-word utterances, respectively.

The average pairwise difference between listeners of the same child was considerably higher for crowdsourced listeners than for in-person listeners, indicating greater variability and reduced reliability among crowdsourced listeners. These metrics showed improvement when each successive in-task quality check criterion was applied, suggesting that interlistener variability can be controlled to some extent. These findings indicate that one approach to managing listener variability in the crowdsourcing context, which inherently has less experimental control, is to apply rigorous in-task quality check criteria to ensure higher quality data. That is, we might compensate for reduced experimental control of listening conditions by being more selective with listener inclusion and requiring a minimum level of performance on preselected trials.

We did not examine the impact of the number of listeners on our findings (beyond considering the performance of a single listener vs. all available listeners for the two sources); however, results of this study suggest that five crowdsourced listeners provided intelligibility data that were more stable (markedly higher ICC values) than data from a single crowdsourced listener (see Table 5). Furthermore, the average intelligibility score of five crowdsourced listeners was about as reliable as an intelligibility score from a single in-person listener, based on comparison of ICC(1, *k*) and ICC(1) values. Thus, another way to manage variability in crowdsourced listeners may be to collect data from more individuals. This finding is consistent with work by Ziegler et al. (2021) who found that listener variability got smaller as the number of listeners increased. One key finding from Ziegler and colleagues was that nine listeners per speaker, where listeners with poor performance were downweighted, provided an optimal estimate of intelligibility that most closely approximated in-person performance for intelligibility of dysarthric speech. It is important to note, however, that the effects of crowdsourced listeners may differ for tasks such as accuracy ratings.

### Crowdsourced Listeners Score Lower but Show the Same Pattern of Results as In-Person Listeners

Consistent with other studies (Cooke & García Lecumberri, 2021), crowdsourced listener results followed the same patterns of age-related change as in-person listener results, but the average intelligibility scores were up to 7 percentage points lower for crowdsourced listeners. There are several potential reasons for this difference between listener sources. First, the laboratory-controlled listening conditions, which reduce distractions and background noise, represent a best-case scenario for measuring intelligibility. The crowdsourced listeners, having to contend with potential distractions and idiosyncratic listening configurations, operate under more of an average-case scenario for measuring intelligibility. Second, in addition to differences in listening environments, there are pertinent differences in listener populations. The demographics of the in-person listeners reflected the university environment: Listeners were younger (20 years old on average), mostly female (74%), and mostly White (82.5%). Crowdsourced listeners in contrast were older (33 years old on average), balanced between genders (47.7% female), and mostly White (73.7%), but more diverse than in-person listeners. In addition, it might be the case that the in-person listeners were systematically better at transcribing child speech. For example, having listeners who live in the same geographic region, and even the same city as most of the speakers, would likely help minimize the effect of dialect differences, broadly speaking.

Rather than focusing on specific population differences between the two sets of listeners, we might instead consider a distribution of listener ability. In-person listeners, being more homogenous overall and less variable, occupy a specific and narrower region of this distribution, while crowdsourced listeners span a broader region of the distribution. For instance, 48 crowdsourced listeners had higher multiword intelligibility scores than their in-person counterparts on the same child. Thus, although mean findings suggest that crowdsourced listeners perform more poorly than in-person listeners, individual listener performance can vary considerably in both directions. One challenge is that in many situations, there is no gold-standard intelligibility metric against which to assess crowdsourced listener performance. Once again, this highlights the importance of having at least five crowdsourced listeners per speaker to ensure the stability of measures so that they are not unduly influenced by high or low performers. Ziegler et al. (2021) examined this issue with different data aggregation approaches, but studies that quantify how many listeners are needed to achieve a stable mean in the context of other data quality control approaches such as in-task screening are needed. Once this is known, we may consider the so-called “crowdsource disadvantage” to be desirable from an ecological validity perspective as it averages over many different kinds of listeners.

### The Largest Discrepancies Between Crowdsourced and In-Person Listeners Occurred for Intelligibility Ranges Between 65% and 83%

From a developmental perspective, the largest difference between listener groups occurred for children between the ages of 2;7 and 5;0 years;months. Perhaps more important than child age, however, was the finding that the intelligibility range corresponding to the largest differences between the two groups of listeners was 65%–83%. Psychometrically, floor and ceiling effects limit intelligibility scores for the least and most intelligible speakers (who are also generally younger and older, respectively); thus, the middle range of speaker intelligibilities and ages has the largest potential for differences between listener types. In this study, we examined typically developing children, all of whom had passed rigorous developmental testing to ensure that speech and language skills were within age expected ranges. While speech intelligibility reductions were present for many children, these reflected maturation effects associated with the course of typical development. However, studies show that listening to degraded speech is cognitively demanding (Peelle, 2018). This midrange of speech intelligibility scores between 65% and 83% might be a range of performance where intelligibility measurement is particularly vulnerable to increased cognitive demands associated with imperfect speech signals, as well as listener and listening environment factors. The finding that there is a disadvantage for crowdsourced listeners within a midrange of intelligibility has important clinical implications for disordered speech and may suggest that crowdsourced listeners should be used with caution. Specifically, there is a risk that crowdsourced intelligibility measures may underrepresent intelligibility scores. Additional studies are needed that systematically evaluate the impact of speaker severity on intelligibility differences between crowdsourced and in-person listeners.

Finally, this study found that when intelligibility is measured using crowdsourced listeners and percentile values based on in-person listener data are derived from the crowdsourced scores, most children will have a considerable reduction in their age percentile results. This means that crowdsourced listener results will underestimate normative intelligibility performance. This finding suggests that norms for speech intelligibility development as reported by Hustad et al. (2021) are most valid with intelligibility scores obtained from in-person listeners. A large-scale replication of the full normative study that includes growth curves and age percentile models based on crowd-sourced listeners may be warranted for future clinical application.

### Clinical Implications

There is a considerable amount of research examining speech intelligibility and other speech perception–related variables that has utilized crowdsourced methods for collecting data from listeners. While crowdsourced listeners are not yet used in clinical practice for measuring intelligibility, such an approach is on the horizon due the efficiency with which data can be collected and the potential ecological validity of drawing from a large and diverse listener pool. The growing use of crowdsourced listeners for measurement of speech variables requires that these methods be well understood in terms of their limitations and the ways that findings are similar to and different from gold-standard in-person intelligibility results.

Findings of the present study suggest that rigorous screening of listeners is important to ensure the validity of results. Furthermore, use of multiple listeners provides data that are more reliable than that from a single listener. Importantly, crowdsourced listeners may have reduced performance relative to in-person listeners in controlled environments, but the same patterns of results seem to hold true regardless of listener modality. A key finding was that individual speakers with moderately reduced intelligibility showed the biggest difference between crowdsourced and in-person listeners. This finding may impact the clinical potential for use of crowdsourcing for speech intelligibility measurement in patient populations such as speakers with dysarthria, speech sound disorders, and so forth, where precise and accurate measurement results have clinical decision making and treatment implications. For example, change in intelligibility due to growth, treatment, or deterioration may not be accurately captured if a speaker moves from moderate severity toward either pole of the severity continuum where the crowdsource disadvantage is reduced. Further research is needed to expand our understanding of how severity of intelligibility reduction influences crowdsourced listener data.

### Limitations and Future Directions

In this study, we examined speech intelligibility of typically developing children who varied in age. By virtue of their age, these children had reduced speech intelligibility. The underlying cause of their intelligibility reductions was related to developmental immaturity, and all children were within age expectations on speech and language measures. Most, if not all, people encounter child speech at some point. Child speech development follows predictable patterns, and there are regularities to speech errors in typical children. As such, findings from the present study may not readily generalize to other cases of reduced speech intelligibility such as those caused by underlying motor impairment, which may lead to error patterns that are irregular, inconsistent, or unpredictable. Studies that examine the impact of crowdsourced listeners on intelligibility of dysarthric speech of varying severity are needed so that we can better understand the pros and cons of this method for collecting intelligibility data. Furthermore, studies should examine how many listeners are necessary to establish stable intelligibility results when rigorous in-task screening criteria are used to ensure data quality. In their study of dysarthric speech, Ziegler et al. (2021) found that nine listeners per speaker were necessary to ensure accurate and valid intelligibility estimates. Future work should evaluate this in combination with other methods of data screening such as in-task criteria thresholds for inclusion.

Research literature using crowdsourced listeners includes many studies where experimental checks for headphone use are incorporated into the research design. There is also considerable literature in which headphone checks are not employed and self-report is used. In the present study, we used self-report of headphone use. This is a limitation of the study and future work should seek to control this important variable more rigorously.

Crowdsourcing is a promising method for collecting large amounts of data from a broad population of listeners at relatively low cost. However, researchers should exercise caution in the interpretation of findings, being careful not to assume that all listener data are created equally. The modality of data collection and the ability to monitor the environment can have a marked impact on findings. Studies should continue to investigate this important methodological approach and how to maximize its power for research on speech intelligibility.

## Data Availability Statement

The codes for all analyses presented in this article are provided in Supplemental Material S1. Data reported in the article are not publicly available due to human subjects' privacy restrictions.

## Supplementary Material

10.1044/2025_JSLHR-25-00391SMS1 Supplemental Material S1 R code used to produce the main statistical findings of the article.
