# Supplementary material for: Does the Use of Crowdsourced Listeners Yield Different Speech Intelligibility Results Than In-Person Listeners for Typically Developing Children?
Source: J Speech Lang Hear Res. 2026 Jan 29;69(2):562–75. doi: 10.1044/2025_JSLHR-25-00391 (PMC12908233; doi:10.1044/2025_JSLHR-25-00391)
Supplement: Supplemental Material S1 [file JSLHR-69-562-s001.pdf]

Supplemental Material S1. R code used to produce the main statistical findings of the article.

Data

Listeners transcribed children's speech, and we measured the children's intelligibilities. Two different sets or sources of listeners were compared (in-person versus crowdsourced). Crowdsourced listeners were presented with an additional set of *fidelity* trials with an adult speaker so that we could compute *fidelity intelligibility* scores. These adult speakers should be easy for a reliable listener to transcribe, so they provide a basis for screening out unreliable listeners. We consider three different data screening scenarios based on whether we apply a fidelity criterion of 0 (i.e., no criterion), .8 or .9. We modeled intelligibility for single-word trials and multiword trials separately.

Sample values from each column of the dataset are given below:

```
library(tidyverse)
data <- targets::tar_read("data_anon")
glimpse(data)
#> Rows: 6,480
#> Columns: 14
#> $ scenario          <chr> "screened_at_00", "screened_at_00", "screened…
#> $ set               <chr> "prolific", "prolific", "prolific", "prolific…
#> $ o_set             <ord> prolific, prolific, prolific, prolific, proli…
#> $ listener          <chr> "l86", "l86", "l86", "l86", "l86", "l86", "l2…
#> $ listener_order    <int> 2, 2, 2, 2, 2, 2, 3, 3, 3, 3, 3, 3, 4, 4, 4, …
#> $ child             <chr> "c34", "c34", "c34", "c34", "c34", "c34", "c3…
#> $ speaker_type      <chr> "adult", "participant", "adult", "participant…
#> $ tocs_type         <chr> "multiword", "multiword", "overall", "overall…
#> $ intelligibility   <dbl> 1.0000000, 0.2166667, 1.0000000, 0.3742690, 1…
#> $ intelligibility2  <dbl> 0.9950000, 0.2166667, 0.9950000, 0.3742690, 0…
#> $ sum_m_word        <dbl> 16, 10, 20, 27, 4, 17, 13, 13, 17, 28, 4, 15,…
#> $ sum_s_word        <dbl> 16, 50, 20, 87, 4, 37, 16, 50, 20, 87, 4, 37,…
#> $ age_months        <dbl> NA, 30, NA, 30, NA, 30, NA, 30, NA, 30, NA, 3…
#> $ age_48            <dbl> NA, -18, NA, -18, NA, -18, NA, -18, NA, -18, …
```

Columns represent the following values:

- `scenario` - Data-screening scenario: `"screened_at_00"` (no data screening), `"screened_at_80"` (at least 80% intelligibility on fidelity trials), and `"screened_at_90"` (at least 90% intelligibility on fidelity trials)
- `set`/`o_set` - Listener type: `"in_person"` or `"prolific"` (crowdsourced). The `o_set` version is an ordered factor used for modeling.
- `listener` - Random ID for the listener who is transcribing the speech samples.
- `listener_order` - Ordering of the listeners (from earliest to latest) within listener type and within each child. This ordering matters because in each data-screening `scenario`, we select the first 5 listeners that meet a data-screening criterion.

- `child` - Random ID for the speaker whose speech samples are being played and transcribed.
- `speaker_type` - Speaker type on intelligibility trials: `"participant"` for the main intelligibility trials (where the speaker is the `child`) or `"adult"` for the fidelity trials used for data-screening.
- `tocs_type` - Intelligibility or speech sample type: `"multiword"` for connected speech samples, `"single-word"` for single words, and `"overall"` for an average across all trials.
- `intelligibility`, `intelligibility2`: Average of trial-level intelligibilities. For each trial, we compute the intelligibility as the proportion of words correctly transcribed on that trials. These proportions are then averaged together to produce intelligibility scores. `intelligibility2` is `intelligibility` squished into the range 0.005–0.995 (for use with beta regression).
- `sum_m_word`, `sum_s_word`: Total number of words correctly transcribed (`sum_m_word`, *matching* words) and total number of words in the speech samples (`sum_s_word`, *sample* words). These aggregations provide a way to compute a different intelligibility measure (proportion of words correctly transcribed), and they are used in a logistic regression model for the single-word trials.
- `age_months`, `age_48`: Age of the `child` in months (`age_months`), and a centered age where 48 months has the value 0 (`age_48`). The `"adult"` `speaker_type` trials don't have an age associated with them.

Each child has 2 in-person listeners and 5 crowdsourced listeners in each data-screening scenario. We can verify these listener counts.

```
data |>
  distinct(child, listener, set, scenario) |>
  count(child, set, scenario, name = "num_listeners") |>
  count(set, scenario, num_listeners, name = "num_children")
#> # A tibble: 6 × 4
#>   set       scenario       num_listeners num_children
#>   <chr>     <chr>                  <int>        <int>
#> 1 in_person screened_at_00             2           60
#> 2 in_person screened_at_80             2           60
#> 3 in_person screened_at_90             2           60
#> 4 prolific  screened_at_00             5           60
#> 5 prolific  screened_at_80             5           60
#> 6 prolific  screened_at_90             5           60
```

We can verify the fidelity intelligibility data screening thresholds in each group.

```
data |>
  filter(
    speaker_type == "adult",
    # average of single-word and connected speech trials
```

```
    tocs_type == "overall"
  ) |>
  group_by(scenario, set) |>
  summarise(
    min_intell = min(intelligibility),
    n_under_80 = sum(intelligibility < .80),
    n_under_90 = sum(intelligibility < .90),
    .groups = "drop"
  )
#> # A tibble: 3 × 5
#>   scenario       set      min_intell n_under_80 n_under_90
#>   <chr>          <chr>         <dbl>      <int>      <int>
#> 1 screened_at_00 prolific      0.625         12         27
#> 2 screened_at_80 prolific      0.812          0         15
#> 3 screened_at_90 prolific      0.906          0          0
```

(These counts slightly differ from a table in the article because that table includes an additional 3 exclusions where an excluded listener’s first replacement also had to be excluded but here we just count the first 5 listeners that satisfy inclusions.)

Intraclass correlation

We report the intraclass correlation statistic as a measure of interrater reliability. We deferred ICC calculation to the psych package (vers. 2.5.3, Revelle, 2024). `pysch::ICC()` requires the data to have one column per “judge” and no grouping variables, so we use `unstack()` (a no-frills `pivot_wider()`) in each `set` × `scenario` × `tocs_type` subgroup, compute the ICCs and extract the relevant values.

```
data |>
  filter(speaker_type == "participant", tocs_type != "overall") |>
  # Number the listeners within each child and each subsample
  group_by(set, scenario, tocs_type, child) |>
  mutate(
    listener_number = seq_along(listener)
  ) |>
  ungroup() |>
  # Compute ICCs in each subsample
  group_by(set, scenario, tocs_type) |>
  reframe(
    datasets = pick(c(listener_number, intelligibility)) |>
      unstack(intelligibility ~ listener_number) |>
      list(),
    iccs = datasets |>
      lapply(psych::ICC, lmer = FALSE) |>
      lapply(getElement, "results")
  ) |>
  unnest(iccs) |>
  filter(type %in% c("ICC2", "ICC2k")) |>
  select(-F, -p, -df1, -df2, -datasets) |>
  mutate(
    set = set |> printy::str_replace_same_as_previous(""),
    scenario = scenario |> printy::str_replace_same_as_previous(""),
    tocs_type = tocs_type |> printy::str_replace_same_as_previous(""),
```

```
  ) |>
  rename(ll = `lower bound`, ul = `upper bound`) |>
  print(n = Inf)
#> # A tibble: 24 × 7
#>    set         scenario        tocs_type     type    ICC    ll    ul
#>    <chr>       <chr>           <chr>         <chr> <dbl> <dbl> <dbl>
#>  1 "in_person" "screened_at_00" "multiword"   ICC2  0.976 0.961 0.986
#>  2 ""          ""              ""            ICC2k 0.988 0.980 0.993
#>  3 ""          ""              "single-word" ICC2  0.913 0.859 0.947
#>  4 ""          ""              ""            ICC2k 0.955 0.924 0.973
#>  5 ""          "screened_at_80" "multiword"   ICC2  0.976 0.961 0.986
#>  6 ""          ""              ""            ICC2k 0.988 0.980 0.993
#>  7 ""          ""              "single-word" ICC2  0.913 0.859 0.947
#>  8 ""          ""              ""            ICC2k 0.955 0.924 0.973
#>  9 ""          "screened_at_90" "multiword"   ICC2  0.976 0.961 0.986
#> 10 ""          ""              ""            ICC2k 0.988 0.980 0.993
#> 11 ""          ""              "single-word" ICC2  0.913 0.859 0.947
#> 12 ""          ""              ""            ICC2k 0.955 0.924 0.973
#> 13 "prolific"  "screened_at_00" "multiword"   ICC2  0.813 0.744 0.872
#> 14 ""          ""              ""            ICC2k 0.956 0.936 0.971
#> 15 ""          ""              "single-word" ICC2  0.681 0.582 0.772
#> 16 ""          ""              ""            ICC2k 0.914 0.875 0.944
#> 17 ""          "screened_at_80" "multiword"   ICC2  0.869 0.818 0.912
#> 18 ""          ""              ""            ICC2k 0.971 0.957 0.981
#> 19 ""          ""              "single-word" ICC2  0.763 0.681 0.835
#> 20 ""          ""              ""            ICC2k 0.941 0.914 0.962
#> 21 ""          "screened_at_90" "multiword"   ICC2  0.888 0.842 0.925
#> 22 ""          ""              ""            ICC2k 0.975 0.964 0.984
#> 23 ""          ""              "single-word" ICC2  0.817 0.749 0.875
#> 24 ""          ""              ""            ICC2k 0.957 0.937 0.972
```

Bayesian generalized additive mixed models

We fit a pair of Bayesian regression models with brms: one for connected speech intelligibility and one for single-word intelligibility. We regressed intelligibility onto age and listener type with by-child random effects of listener type. We only examined `"participant"` intelligibility scores (the fidelity trials were only used for data-screening). These models had repeated measures with 1 intelligibility score per listener and 2 or 5 listeners per child.

The same basic regression setup was used to fit each model, so we describe the first model in more detail.

Connected speech model

We used beta regression for the connected speech model. brms uses the mean-precision (μ, ϕ) parameterization of the beta distribution where the mean is a proportion and the precision is a positive number. We used the default link functions: logit for the mean (so the model for the mean works on the log-odds scale) and log for the precision.

```
targets::tar_load("model_stocs_difference_smooth")
model_stocs_difference_smooth |>
```

```
  family() |>
  _[c("family", "dpars", "link", "link_phi")] |>
  str()
#> List of 4
#>  $ family  : chr "beta"
#>  $ dpars   : chr [1:2] "mu" "phi"
#>  $ link    : chr "logit"
#>  $ link_phi: chr "log"
```

Beta regression does not allow proportions of 0 or 1, so these floor and ceiling values were squished into the range 0.005 and .995.

```
y <- model_stocs_difference_smooth$data$intelligibility2
range(y)
#> [1] 0.08333333 0.99500000
sum(y == .005)
#> [1] 0
sum(y == .995)
#> [1] 6
```

The regression model in brms syntax is given below:

```
formula(model_stocs_difference_smooth)
#> intelligibility2 ~ set + s(age_48) + s(age_48, by = o_set) + (set | child)
#> phi ~ set + age_48
```

For the mean model, we have a parametric effect of listener type (`set`) and by-child random effects of listener type (`(set | child)`). We also have a thin-plate spline smooth of age (`s(age_48)`) and a *difference smooth* of age for the two listener types. Supplying an ordered factor (`o_set`) causes mgcv/brms to compute a difference smooth.

Below we plot the baseline smooth (in-person listeners) and the difference smooth (in-person minus crowdsourced listeners). There is not a clear difference in the effects of age on intelligibility in the smooths between the two listener groups (on the logit scale):

```
library(ggplot2)
library(patchwork)

cs1 <- model_stocs_difference_smooth |>
  brms::conditional_smooths("s(age_48)") |>
  # i.e., return the plot object but don't display it
  plot(plot = FALSE)
#> Loading required namespace: rstan

cs2 <- model_stocs_difference_smooth |>
  brms::conditional_smooths(
    "s(age_48, by = o_set)",
    int_conditions = list(o_set = "prolific")
  ) |>
  plot(plot = FALSE)

cs1[[1]] + ylim(-2.75, 2.75) + cs2[[1]] + ylim(-2.75, 2.75)
```

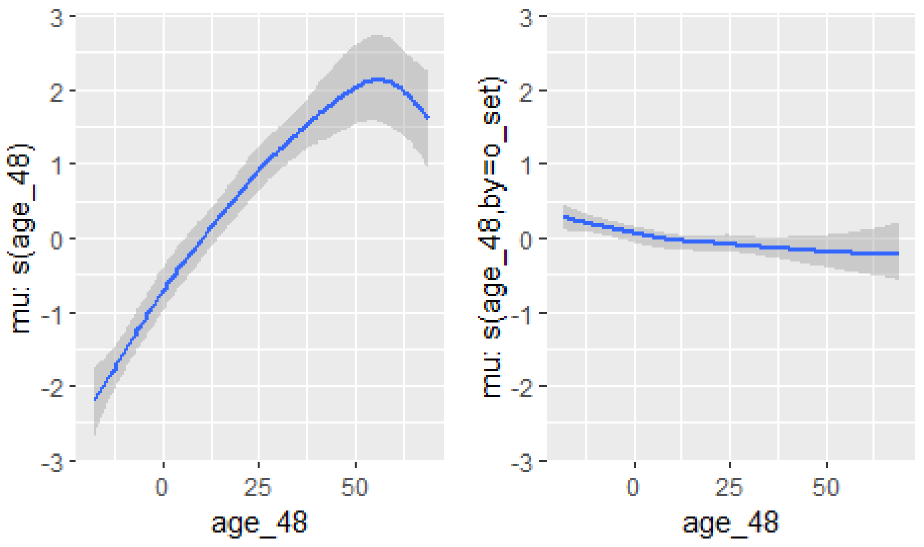

Marginal means

Our visualizations used marginal means from each model. That is, a mixed effects model’s fixed effects estimates a conditional mean: It is the expected intelligibility score for a participant whose random effect values have all been set to 0. This conditional mean is not the same as the *marginal* or population mean, so we have take additional steps to compute this marginal mean. Namely, we simulate new children based on the model (sample new random effect values) and then average (or *marginalize*) over them.

We used the following recipe to compute marginal means.

```
# 0. Prepare a prediction grid for a new out-of-sample participant
newdata <- targets::tar_read(newdata_stocs)

compute_tocs_marginal_means <- function(
    newdata,
    model,
    n = 1000,
    seed = NULL
) {
  # This model has 2 correlated random effects, so we need to draw n
  # participants from the model's random-effect variance-covariance matrix
  # on each posterior draw.

  if (!is.null(seed)) withr::local_seed(as.integer(seed))

  # 1. Get conditional means (logits) when random effects are zeroed out
  data_linpreds <- newdata |>
    tidybayes::add_linpred_rvars(
      model,
      ndraws = posterior::ndraws(model),
      re_formula = NA
    ) |>
    dplyr::select(-o_set, -control_file) |>
```

```
    tidyr::pivot_wider(names_from = "set", values_from = ".linpred")

  # 2. Get random effect variance-covariance matrix.
  cov <- model |>
    brms::VarCorr(summary = FALSE) |>
    _$child$cov |>
    posterior::rvar()
  means <- rep(0, ncol(cov))

  # 3. Simulate n new random effects (children) on each posterior draw.
  new_children <- posterior::rdo(mvtnorm::rmvnorm(n, means, cov))

  # 4. Add conditional means to simulated random effects, convert to
  #    proportion scale and average the proportions within each
  #    posterior draw

  # Handle random effects like (0 + set | child) where the marginal is
  #   `fixed effects + new_children[, <1 or 2>]`
  if (all(rownames(cov) == c("setin_person", "setprolific"))) {
    data_marginal_preds <- data_linpreds |>
      dplyr::rowwise() |>
      dplyr::mutate(
        in_person.conditional = .data$in_person |>
          brms::inv_logit_scaled(),
        prolific.conditional = .data$prolific |>
          brms::inv_logit_scaled(),
        in_person.marginal = (.data$in_person + new_children[, 1]) |>
          brms::inv_logit_scaled() |>
          posterior::rvar_mean(),
        prolific.marginal = (.data$prolific + new_children[, 2]) |>
          brms::inv_logit_scaled() |>
          posterior::rvar_mean()
      ) |>
      dplyr::ungroup() |>
      dplyr::mutate(
        diff.conditional = in_person.conditional - prolific.conditional,
        diff.marginal = in_person.marginal - prolific.marginal
      ) |>
      dplyr::select(age_48, ends_with("marginal"), ends_with("conditional"))

  }

  # Handle random effects like (set | child) where the marginal is
  #   `fixed effect + new_children[, 1] + <0 or new_children[, 2]`
  if (all(rownames(cov) == c("Intercept", "setprolific"))) {
    data_marginal_preds <- data_linpreds |>
      dplyr::rowwise() |>
      dplyr::mutate(
        in_person.conditional = .data$in_person |>
          brms::inv_logit_scaled(),
        prolific.conditional = .data$prolific |>
          brms::inv_logit_scaled(),
        in_person.marginal = (.data$in_person + new_children[, 1]) |>
```

```
          brms::inv_logit_scaled() |>
          posterior::rvar_mean(),
        prolific.marginal =
          (.data$prolific + new_children[, 1] + new_children[, 2]) |>
          brms::inv_logit_scaled() |>
          posterior::rvar_mean()
      ) |>
      dplyr::ungroup() |>
      dplyr::mutate(
        diff.conditional = in_person.conditional - prolific.conditional,
        diff.marginal = in_person.marginal - prolific.marginal
      ) |>
      dplyr::select(age_48, ends_with("marginal"), ends_with("conditional"))
  }

  # 5. Reshape to have one row per set
  data_marginal_preds |>
    tidyr::pivot_longer(
      cols = c(-age_48),
      names_pattern = "(.+)(.conditional|.marginal)",
      names_to = c("set", "type"),
      values_to = "value"
    ) |>
    tidyr::pivot_wider(names_from = type, values_from = value) |>
    # unnested form is faster/smaller to write to disk
    tidybayes::unnest_rvars()
}
```

Because of the long computation time, we instead read in and preview a precomputed copy of the marginal means.

```
data_stocs_marginal_means <- targets::tar_read(data_stocs_marginal_means)
data_stocs_marginal_means
#> # A tibble: 1,056,000 × 9
#>    age_48 set      .marginal .conditional .chain .iteration .draw outcome
#>     <dbl> <chr>        <dbl>        <dbl>  <int>      <int> <int> <chr>
#>  1    -18 in_per…      0.462        0.456      1          1     1 stocs
#>  2    -18 in_per…      0.510        0.515      1          2     2 stocs
#>  3    -18 in_per…      0.509        0.502      1          3     3 stocs
#>  4    -18 in_per…      0.508        0.513      1          4     4 stocs
#>  5    -18 in_per…      0.495        0.493      1          5     5 stocs
#>  6    -18 in_per…      0.390        0.378      1          6     6 stocs
#>  7    -18 in_per…      0.411        0.403      1          7     7 stocs
#>  8    -18 in_per…      0.437        0.421      1          8     8 stocs
#>  9    -18 in_per…      0.410        0.409      1          9     9 stocs
#> 10    -18 in_per…      0.411        0.402      1         10    10 stocs
#> # ℹ 1,055,990 more rows
#> # ℹ 1 more variable: level <chr>
```

From these conditional means, it is straightforward to compute growth curves presented in the article:

```
ggplot(data_stocs_marginal_means) +
  aes(x = age_48, y = .marginal) +
  ggdist::stat_lineribbon(
    aes(group = set, fill = set),
    .width = .95,
    alpha = .5
  )
```

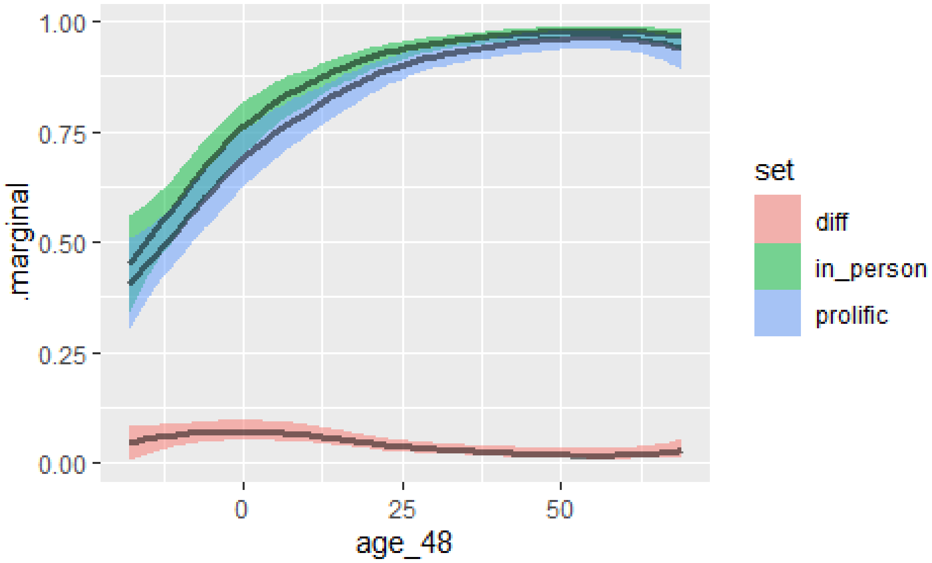

Effect of intelligibility on listener type difference

For the plot of the in-person listener advantage versus in-person intelligibility, we fix the in-person intelligibility to a narrow range of values (i.e., we bin them) and gather all of the intelligibility difference scores for that range of values.

For example, here are all the rows in the marginal means dataframe where the in-person marginal means that are binned to .65.

```
d <- data_stocs_marginal_means |>
  select(-.conditional) |>
  filter(set != "prolific") |>
  tidyr::pivot_wider(names_from = set, values_from = .marginal) |>
  ungroup()

d_at_65 <- d |>
  mutate(
    in_person = plyr::round_any(in_person, .025)
  ) |>
  filter(in_person == .65)

d_at_65
#> # A tibble: 5,763 × 8
#>    age_48 .chain .iteration .draw outcome level in_person   diff
#>     <dbl>  <int>      <int> <int> <chr>   <chr>     <dbl>  <dbl>
#>  1    -18      1       3848  3848 stocs   i90        0.65 0.0400
```

```
#>  2    -17       1        1189  1189 stocs   i90         0.65 0.0608
#>  3    -17       1        3848  3848 stocs   i90         0.65 0.0436
#>  4    -16       1         477   477 stocs   i90         0.65 0.0425
#>  5    -16       1        1189  1189 stocs   i90         0.65 0.0614
#>  6    -16       1        3848  3848 stocs   i90         0.65 0.0472
#>  7    -15       1         477   477 stocs   i90         0.65 0.0459
#>  8    -15       1         478   478 stocs   i90         0.65 0.0793
#>  9    -15       1        1189  1189 stocs   i90         0.65 0.0620
#> 10    -15       1        3848  3848 stocs   i90         0.65 0.0509
#> # ℹ 5,753 more rows
```

There are 5763 ages × posterior draws where the marginal mean is binned to this value. These include predictions from different ages and different amounts of listener type differences (`diff`). We can look at the distribution of these listener type differences.

```
hist(d_at_65$diff)
```

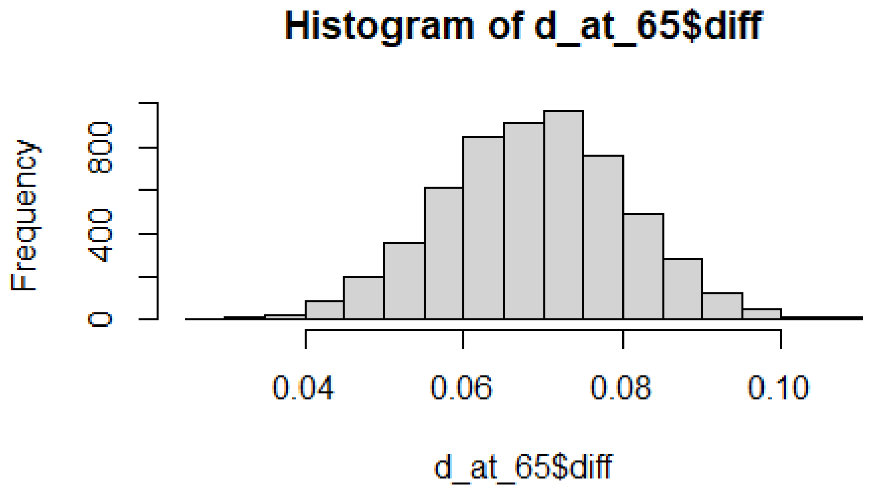

This distribution, for each difference, are presented in the article. We used the general recipe given below. The main details are that the differences 1) we exclude any bins with fewer than 200 posterior draws and 2) within each bin the differences within a posterior draw are averaged together.

```
# Bin the in-person scores and count the differences in each bin. Discard
# any bin with fewer than 200 posterior total draws.
d_intervals <- d |>
  mutate(
    in_person = plyr::round_any(in_person, .025)
  ) |>
  group_by(outcome, level, in_person) |>
  mutate(n_draws = n()) |>
  ungroup() |>
  filter(n_draws > 200) |>
  # If a posterior draw hits an in-person intelligibility bin multiple
  # times, because a curve plateaus or horseshoes, take the average
```

```
  # of the differences for each draw. Now each posterior draw
  # can only have one vote.
  group_by(outcome, level, in_person, .draw) |>
  summarise(
    diff = mean(diff),
    .group = "drop"
  ) |>
  # For each in-person intelligibility bin, get median and 95% interval.
  group_by(outcome, level, in_person) |>
  ggdist::median_qi(diff)
#> `summarise()` has grouped output by 'outcome', 'level', 'in_person'.
#> You can override using the `.groups` argument.

d_intervals
#> # A tibble: 27 × 9
#>    outcome level in_person   diff   .lower .upper .width .point
#>    <chr>   <chr>     <dbl>  <dbl>    <dbl>  <dbl>  <dbl> <chr>
#>  1 stocs   i90       0.35  0.0336 -0.00834 0.0618   0.95 median
#>  2 stocs   i90       0.375 0.0370 -0.00256 0.0666   0.95 median
#>  3 stocs   i90       0.4   0.0393  0.00313 0.0700   0.95 median
#>  4 stocs   i90       0.425 0.0417  0.00600 0.0735   0.95 median
#>  5 stocs   i90       0.45  0.0456  0.0113  0.0773   0.95 median
#>  6 stocs   i90       0.475 0.0493  0.0163  0.0806   0.95 median
#>  7 stocs   i90       0.5   0.0528  0.0210  0.0827   0.95 median
#>  8 stocs   i90       0.525 0.0561  0.0257  0.0843   0.95 median
#>  9 stocs   i90       0.55  0.0594  0.0312  0.0858   0.95 median
#> 10 stocs   i90       0.575 0.0624  0.0358  0.0872   0.95 median
#> # ℹ 17 more rows
#> # ℹ 1 more variable: .interval <chr>

ggplot(d_intervals) +
  aes(x = in_person) +
  geom_ribbon(aes(ymin = .lower, ymax = .upper), alpha = .2) +
  geom_line(aes(y = diff))
```

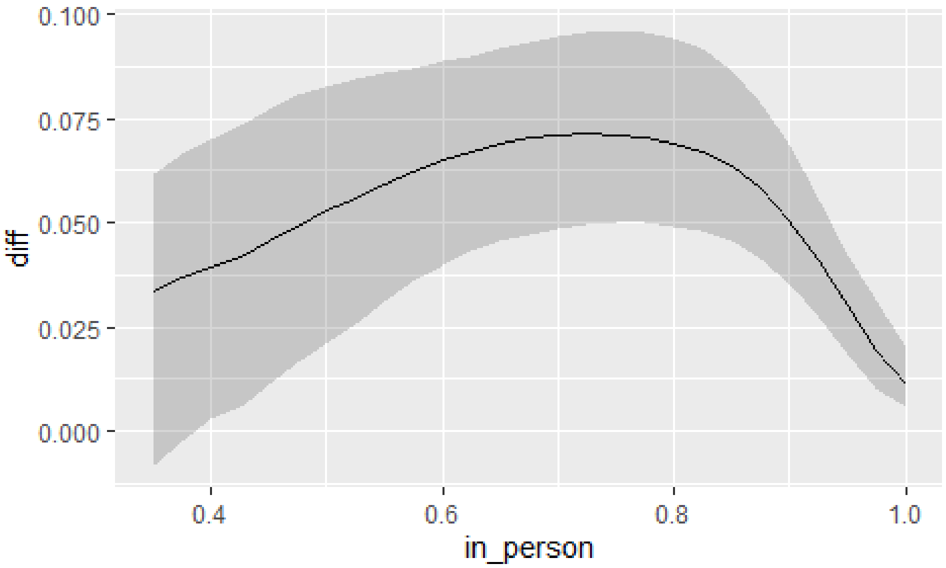

Single-word intelligibility

The single-word intelligibility model was completely analogous to the multiword model except that it was used logistic regression model (a binomial family with logit link).

```
targets::tar_load("model_wtocs_difference_smooth")
model_wtocs_difference_smooth |>
  family() |>
  _[c("family", "dpars", "link")] |>
  str()
#> List of 3
#>  $ family: chr "binomial"
#>  $ dpars : chr "mu"
#>  $ link  : chr "logit"
```

The binomial family estimates a number of successes in a number of trials. The brms `trials()` syntax capture those values in the lefthand side of the regression formula:

```
formula(model_wtocs_difference_smooth)
#> sum_m_word | trials(sum_s_word) ~ set + s(age_48) + s(age_48, by = o_set) + (set
| child)
```

Recall that `sum_m_word` is the total number of words correctly transcribed by a listener and `sum_s_word` is the total number of words presented to the listener.

This model also used a difference smooth, and we can visualize the two smooths as we did above.

```
cs1 <- model_wtocs_difference_smooth |>
  brms::conditional_smooths("s(age_48)") |>
  # i.e., return the plot object but don't display it
  plot(plot = FALSE)
#> Setting all 'trials' variables to 1 by default if not specified otherwise.

cs2 <- model_wtocs_difference_smooth |>
  brms::conditional_smooths(
    "s(age_48, by = o_set)",
    int_conditions = list(o_set = "prolific")
  ) |>
  plot(plot = FALSE)
#> Setting all 'trials' variables to 1 by default if not specified otherwise.

cs1[[1]] + ylim(-2.75, 2.75) + cs2[[1]] + ylim(-2.75, 2.75)
```

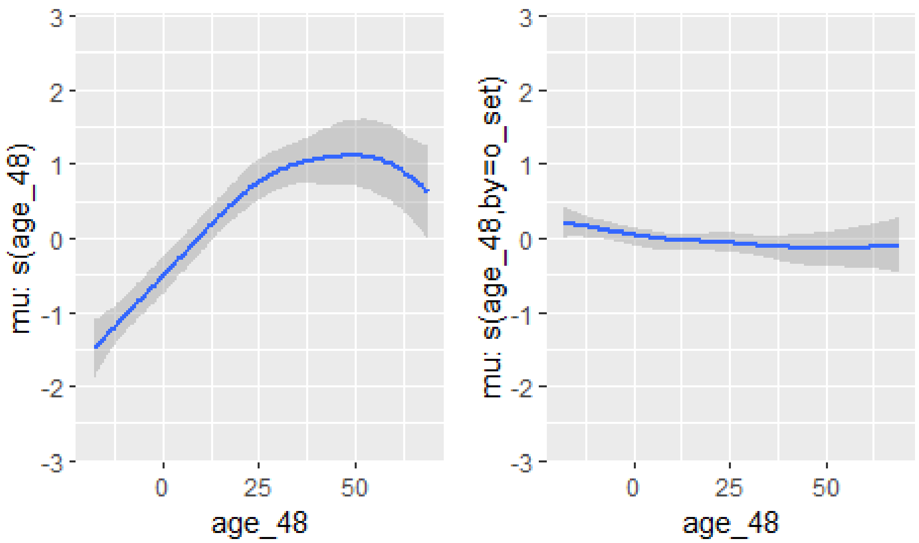

The marginal means computation and differences-by-in-person-intelligibility computation used the same recipes (and code) as outlined above.

Model comparisons

Do we need the difference smooth in each of these models? The right panel of the marginal smooths mostly shows a horizontal line hovering around 0, suggesting a limited difference. Model comparison with approximate leave-one-out (LOO) techniques (Vehtari et al., 2017), on the other hand, does not strongly favor the simpler shared smooth model over the model with a baseline smooth and difference smooth.

```
targets::tar_load(model_stocs_difference_smooth)
targets::tar_load(model_stocs_shared_smooth)
targets::tar_load(model_wtocs_difference_smooth)
targets::tar_load(model_wtocs_shared_smooth)

brms::loo_compare(
  model_wtocs_shared_smooth,
  model_wtocs_difference_smooth
) |>
  print(simplify = FALSE)
#>                               elpd_diff se_diff elpd_loo se_elpd_loo
#> model_wtocs_difference_smooth     0.0       0.0 -1066.3     27.8
#> model_wtocs_shared_smooth        -2.1       2.4 -1068.4     28.1
#>                               p_loo   se_p_loo looic   se_looic
#> model_wtocs_difference_smooth   87.5     8.5   2132.6    55.6
#> model_wtocs_shared_smooth       90.0     8.8   2136.7    56.2

brms::loo_compare(
  model_stocs_shared_smooth,
  model_stocs_difference_smooth
) |>
  print(simplify = FALSE)
```

```
#>                                 elpd_diff se_diff elpd_loo se_elpd_loo
#> model_stocs_difference_smooth     0.0       0.0    680.5     25.4
#> model_stocs_shared_smooth        -5.0       3.1    675.5     26.5
#>                                 p_loo    se_p_loo looic    se_looic
#> model_stocs_difference_smooth    65.5      5.3  -1361.1     50.8
#> model_stocs_shared_smooth        65.7      5.5  -1351.1     53.0
```

We interpret this output by looking at `elpd_loo` (expected log predictive density). Higher values indicate better expected predictive performance on out of sample data. The difference in ELPD between each model and the highest ELPD model (i.e., first row) is given in `elpd_diff`. We need to interpret these differences in light of the standard error `se_diff`. Neither of the differences reported above is greater than 2-SEs in magnitude, so there is not a statistically clear difference between the models in terms of predictive performance.

We also note that leave-one-out comparison is not ideal for repeated-measures data but leave-one-group-out techniques are computationally prohibitive.

Sampling details

Hamilitonian Monte Carlo sampling diagnostics indicated no major issues. There were no divergent transitions, the max treedepth on the iterations did not reach the treedepth limit, and the so-called estimated fraction of missing information statistic was greater than .3 on every chain.

```
get_hmc_details <- function(model, ...) {
  df <- brms::nuts_params(model, ...)
  df$model <- as.character(substitute(model))
  df$limit_treedepth <- model$stan_args$control$max_treedepth %||% 10
  tidyr::pivot_wider(
    data = df,
    names_from = "Parameter",
    values_from = "Value"
  )
}

bind_rows(
  get_hmc_details(model_stocs_difference_smooth),
  get_hmc_details(model_wtocs_difference_smooth)
) |>
  group_by(model, chain = Chain) |>
  summarise(
    n_draws = n(),
    efmi = mean(diff(energy__) ^ 2) / var(energy__),
    n_divergent = sum(divergent__),
    max_depth = max(treedepth__),
    limit_depth = unique(limit_treedepth),
    .groups = "drop"
  )
#> # A tibble: 8 × 7
#>   model             chain n_draws  efmi n_divergent max_depth limit_depth
#>   <chr>             <int>   <int> <dbl>       <dbl>     <dbl>       <dbl>
#> 1 model_stocs_dif…      1    1000 0.659           0         9          10
```

```
#> 2 model_stocs_dif…     2    1000 0.705           0         9          10
#> 3 model_stocs_dif…     3    1000 0.830           0         9          10
#> 4 model_stocs_dif…     4    1000 0.673           0         9          10
#> 5 model_wtocs_dif…     1    1000 0.683           0        10          15
#> 6 model_wtocs_dif…     2    1000 0.773           0         9          15
#> 7 model_wtocs_dif…     3    1000 0.696           0         9          15
#> 8 model_wtocs_dif…     4    1000 0.773           0         9          15
```

The Rhat and effective sample size statistics were also satisfactory (Rhats <= 1.01, ESS > 100 per chain, or 400):

```
get_convergence_stats <- function(model) {
  df <- posterior::summarise_draws(
    model,
    posterior::default_convergence_measures()
  )
  df$model <- as.character(substitute(model))
  df
}

bind_rows(
  get_convergence_stats(model_stocs_difference_smooth),
  get_convergence_stats(model_wtocs_difference_smooth)
) |>
  group_by(model) |>
  summarise(
    model = "model_stocs_difference_smooth",
    max_rhat = max(rhat),
    min_ess_bulk = min(ess_bulk),
    min_ess_tail = min(ess_tail)
  )
#> # A tibble: 2 × 4
#>   model                         max_rhat min_ess_bulk min_ess_tail
#>   <chr>                            <dbl>        <dbl>        <dbl>
#> 1 model_stocs_difference_smooth     1.01         724.         968.
#> 2 model_stocs_difference_smooth     1.01         470.        1220.
```

Prior description

Because these models estimate means on the logit scale, we used weakly informative priors of Normal(0, 1) for the regression coefficients and standard deviations of the random effects. Because mgcv transforms a spline basis to absorb constraints and penalty terms in sophisticated ways, appropriate scale values for the smooth terms are not clear a priori. Therefore, we used wider Normal(0, 2) priors for the SDs of the smoothing coefficients.

We used a weakly informative LKJ(2) prior on the correlations in the random-effect matrix. This prior pulls probability mass away from boundary correlations (1 or −1).

For the beta regression model, we also estimated a phi parameter with a log link function and as a result, the effect of age on the precision on the outcome scale is multiplicative. Therefore, we used a weakly informative Normal(0, 2) prior.

Any other unspecified priors used brms defaults.

```
model_stocs_difference_smooth$prior
#>                prior     class                      coef group resp dpar
#>         normal(0, 1)         b
#>         normal(0, 1)         b sage_48:o_setprolific_1
#>         normal(0, 1)         b                 sage_48_1
#>         normal(0, 1)         b               setprolific
#>         normal(0, 2)         b                                         phi
#>         normal(0, 2)         b                    age_48               phi
#>         normal(0, 2)         b               setprolific               phi
#>         normal(0, 1) Intercept
#>  student_t(3, 0, 2.5) Intercept                                        phi
#>  lkj_corr_cholesky(2)         L
#>  lkj_corr_cholesky(2)         L                            child
#>         normal(0, 1)        sd
#>         normal(0, 1)        sd                             child
#>         normal(0, 1)        sd                 Intercept child
#>         normal(0, 1)        sd               setprolific child
#>         normal(0, 2)       sds
#>         normal(0, 2)       sds                 s(age_48)
#>         normal(0, 2)       sds   s(age_48, by = o_set)
#>  nlpar lb ub       source
#>                      user
#>              (vectorized)
#>              (vectorized)
#>              (vectorized)
#>                      user
#>              (vectorized)
#>              (vectorized)
#>                      user
#>                   default
#>                      user
#>              (vectorized)
#>        0             user
#>        0     (vectorized)
#>        0     (vectorized)
#>        0     (vectorized)
#>        0             user
#>        0     (vectorized)
#>        0     (vectorized)

model_wtocs_difference_smooth$prior
#>                prior     class                      coef group resp dpar
#>         normal(0, 1)         b
#>         normal(0, 1)         b sage_48:o_setprolific_1
#>         normal(0, 1)         b                 sage_48_1
#>         normal(0, 1)         b               setprolific
#>         normal(0, 1) Intercept
#>  lkj_corr_cholesky(2)         L
#>  lkj_corr_cholesky(2)         L                            child
#>         normal(0, 1)        sd
#>         normal(0, 1)        sd                             child
#>         normal(0, 1)        sd                 Intercept child
```

```
#>          normal(0, 1)       sd             setprolific child
#>          normal(0, 2)      sds
#>          normal(0, 2)      sds                 s(age_48)
#>          normal(0, 2)      sds   s(age_48, by = o_set)
#>  nlpar lb ub       source
#>                      user
#>              (vectorized)
#>              (vectorized)
#>              (vectorized)
#>                      user
#>                      user
#>              (vectorized)
#>         0            user
#>         0    (vectorized)
#>         0    (vectorized)
#>         0    (vectorized)
#>         0            user
#>         0    (vectorized)
#>         0    (vectorized)
```

Software details

Analyses were conducted in the R programming language (vers. 4.5.0, R Core Team, 2025). Models were fit using the Stan programming language (vers. 2.36.0, Stan Development Team, 2024) via the brms (vers. 2.22.0, Bürkner, 2017) and cmdstanr (vers. 0.9.0, Gabry et al., 2024) R packages. The implementation of smoothing splines relies on mgcv (vers. 1.9.1, Wood, 2017). Handling the posterior samples was greatly simplified by tidybayes (vers. 3.0.7, Kay, 2024b) and ggdist (vers. 3.3.3, Kay, 2024a).

Because we rely on reading in cached models, it's important to ask the models for their software versions as well:

```
model_stocs_difference_smooth$version |>
  lapply(as.character) |>
  str()
#> List of 5
#>  $ brms       : chr "2.22.0"
#>  $ rstan      : chr "2.32.7"
#>  $ stanHeaders: chr "2.32.10"
#>  $ cmdstanr   : chr "0.9.0"
#>  $ cmdstan    : chr "2.36.0"

model_wtocs_difference_smooth$version |>
  lapply(as.character) |>
  str()
#> List of 5
#>  $ brms       : chr "2.22.0"
#>  $ rstan      : chr "2.32.7"
#>  $ stanHeaders: chr "2.32.10"
#>  $ cmdstanr   : chr "0.9.0"
#>  $ cmdstan    : chr "2.36.0"
```

Full Model Summaries

For completeness, here are the full model summaries, but note that these are generalized linear models with nonlinear link functions so it is difficulty to interpret individual coefficients in isolation. Note also that the quantities for the smoothing spline SDs and linear effects from each smooth are generally uninterpretable because of several matrix transformations. Thus, these models are best understood through the expectations or predictions computed from them.

Multiword intelligibility:

```
summary(model_stocs_difference_smooth, priors = TRUE)
#>  Family: beta
#>   Links: mu = logit; phi = log
#> Formula: intelligibility2 ~ set + s(age_48) + s(age_48, by = o_set) + (set |
child)
#>          phi ~ set + age_48
#>    Data: structure(list(scenario = c("screened_at_90", "scr (Number of
observations: 420)
#>   Draws: 4 chains, each with iter = 2000; warmup = 1000; thin = 1;
#>          total post-warmup draws = 4000
#> 
#> Priors:
#> b ~ normal(0, 1)
#> b_phi ~ normal(0, 2)
#> Intercept ~ normal(0, 1)
#> Intercept_phi ~ student_t(3, 0, 2.5)
#> L ~ lkj_corr_cholesky(2)
#> <lower=0> sd ~ normal(0, 1)
#> <lower=0> sds ~ normal(0, 2)
#> 
#> Smoothing Spline Hyperparameters:
#>                               Estimate Est.Error l-95% CI u-95% CI Rhat
#> sds(sage_48_1)                    1.68      0.73     0.68     3.48 1.00
#> sds(sage_48o_setprolific_1)       0.33      0.30     0.01     1.15 1.00
#>                               Bulk_ESS Tail_ESS
#> sds(sage_48_1)                    1220     2098
#> sds(sage_48o_setprolific_1)       1297     1956
#> 
#> Multilevel Hyperparameters:
#> ~child (Number of levels: 60)
#>                              Estimate Est.Error l-95% CI u-95% CI Rhat
#> sd(Intercept)                    0.67      0.07     0.54     0.82 1.00
#> sd(setprolific)                  0.13      0.05     0.02     0.23 1.00
#> cor(Intercept,setprolific)      -0.66      0.23    -0.95    -0.07 1.00
#>                              Bulk_ESS Tail_ESS
#> sd(Intercept)                     944     1724
#> sd(setprolific)                  1372      968
#> cor(Intercept,setprolific)       2958     1876
#> 
#> Regression Coefficients:
#>                          Estimate Est.Error l-95% CI u-95% CI Rhat
#> Intercept                    1.97      0.09     1.78     2.15 1.00
```

```
#> phi_Intercept                 4.89      0.17     4.55     5.23 1.00
#> setprolific                  -0.46      0.05    -0.56    -0.37 1.00
#> phi_setprolific              -1.57      0.19    -1.94    -1.19 1.00
#> phi_age_48                    0.00      0.00    -0.00     0.01 1.00
#> sage_48_1                     0.74      0.96    -1.19     2.62 1.00
#> sage_48:o_setprolific_1      -0.55      0.54    -1.66     0.59 1.00
#>                           Bulk_ESS Tail_ESS
#> Intercept                      750     1388
#> phi_Intercept                 3153     2753
#> setprolific                   3647     3069
#> phi_setprolific               3588     3117
#> phi_age_48                    6466     3636
#> sage_48_1                     5196     2582
#> sage_48:o_setprolific_1       2719     2599
#>
#> Draws were sampled using sample(hmc). For each parameter, Bulk_ESS
#> and Tail_ESS are effective sample size measures, and Rhat is the potential
#> scale reduction factor on split chains (at convergence, Rhat = 1).
```

Single word intelligibility:

```
summary(model_wtocs_difference_smooth, priors = TRUE)
#>  Family: binomial
#>   Links: mu = logit
#> Formula: sum_m_word | trials(sum_s_word) ~ set + s(age_48) + s(age_48, by =
o_set) + (set | child)
#>    Data: structure(list(scenario = c("screened_at_90", "scr (Number of
observations: 420)
#>   Draws: 4 chains, each with iter = 2000; warmup = 1000; thin = 1;
#>          total post-warmup draws = 4000
#>
#> Priors:
#> b ~ normal(0, 1)
#> Intercept ~ normal(0, 1)
#> L ~ lkj_corr_cholesky(2)
#> <lower=0> sd ~ normal(0, 1)
#> <lower=0> sds ~ normal(0, 2)
#>
#> Smoothing Spline Hyperparameters:
#>                               Estimate Est.Error l-95% CI u-95% CI Rhat
#> sds(sage_48_1)                    1.23      0.60     0.48     2.72 1.00
#> sds(sage_48o_setprolific_1)       0.36      0.36     0.01     1.39 1.00
#>                               Bulk_ESS Tail_ESS
#> sds(sage_48_1)                    1664     2662
#> sds(sage_48o_setprolific_1)       1597     1930
#>
#> Multilevel Hyperparameters:
#> ~child (Number of levels: 60)
#>                              Estimate Est.Error l-95% CI u-95% CI Rhat
#> sd(Intercept)                    0.60      0.07     0.47     0.75 1.00
#> sd(setprolific)                  0.14      0.08     0.01     0.31 1.01
#> cor(Intercept,setprolific)       0.05      0.35    -0.60     0.76 1.00
#>                              Bulk_ESS Tail_ESS
#> sd(Intercept)                    1712     2513
```

```
#> sd(setprolific)                 470     1474
#> cor(Intercept,setprolific)     2780     1731
#>
#> Regression Coefficients:
#>                            Estimate Est.Error l-95% CI u-95% CI Rhat
#> Intercept                      1.39      0.09     1.21     1.56 1.00
#> setprolific                   -0.30      0.05    -0.40    -0.19 1.00
#> sage_48_1                      0.47      0.93    -1.39     2.26 1.00
#> sage_48:o_setprolific_1       -0.30      0.54    -1.35     0.90 1.00
#>                            Bulk_ESS Tail_ESS
#> Intercept                       985     1779
#> setprolific                    6484     3400
#> sage_48_1                      4254     3055
#> sage_48:o_setprolific_1        3689     2544
#>
#> Draws were sampled using sample(hmc). For each parameter, Bulk_ESS
#> and Tail_ESS are effective sample size measures, and Rhat is the potential
#> scale reduction factor on split chains (at convergence, Rhat = 1).
```

Modeling functions

The following code block shows the modeling workflow we used to fit these models. Each family of models has a single function. A user passes in a dataset, a model `flavor`, an optional random number seed and optional `tag`. The `flavor` is used to look up the appropriate model formula, and the `tag` is used to differentiate two models with different datasets but the same flavor.

```
fit_stocs_model <- function(
    data,
    flavor,
    tag = "",
    seed = NA,
    use_reloo = FALSE
) {
  formulas <- list(
    stocs_shared_smooth = bf(
      intelligibility2 ~ set + s(age_48) +
        (set | child),
      phi ~ set + age_48,
      family = Beta
    ),
    stocs_difference_smooth = bf(
      intelligibility2 ~
        set +
        s(age_48) +
        s(age_48, by = o_set) +
        (set | child),
      phi ~ set + age_48,
      family = Beta
    )
  )

  formula <- formulas[[flavor]]
```

```
  loo_slug <- ifelse(use_reloo, "_reloo", "")
  file <- file.path(
    "models", paste0(flavor, tag, loo_slug)
  )

  prior <- if (has_random_slope_correlation(formula, data)) {
    lookup_brms_priors("logit_phi_w_random_slopes")
  } else {
    lookup_brms_priors("logit_phi_w_random_intercepts")
  }

  args <- brm_args(
    formula = formula,
    data = data,
    prior = prior,
    file = file,
    seed = seed,
    adapt_delta = .98,
    iter = 2000
  )
  model <- do.call(brms::brm, args)

  add_loo_criterion(model, use_reloo)
}

fit_wtocs_model <- function(
    data,
    flavor,
    tag = "",
    seed = NA,
    use_reloo = FALSE
) {
  formulas <- list(
    wtocs_shared_smooth = bf(
      sum_m_word | trials(sum_s_word) ~
        set +
        s(age_48) +
        (set | child),
      family = binomial
    ),
    wtocs_difference_smooth = bf(
      sum_m_word | trials(sum_s_word) ~
        set +
        s(age_48) +
        s(age_48, by = o_set) +
        (set | child),
      family = binomial
    ),
    wtocs_difference_smooth_simple_ranef = bf(
      sum_m_word | trials(sum_s_word) ~
        set +
        s(age_48) +
        s(age_48, by = o_set) +
        (0 + set | child),
```

```
      family = binomial
    )
  )

  formula <- formulas[[flavor]]
  loo_slug <- ifelse(use_reloo, "_reloo", "")
  file <- file.path(
    "models", paste0(flavor, tag, loo_slug)
  )

  prior <- if (has_random_slope_correlation(formula, data)) {
    lookup_brms_priors("logit_w_random_slopes")
  } else {
    lookup_brms_priors("logit_w_random_intercepts")
  }

  args <- brm_args(
    formula = formula,
    data = data,
    prior = prior,
    file = file,
    seed = seed,
    adapt_delta = .99,
    max_treedepth = 15
  )
  model <- do.call(brms::brm, args)

  add_loo_criterion(model, use_reloo)
}

lookup_brms_priors <- function(set = "logit_w_random_intercepts") {
  l <- list(
    logit_w_random_intercepts = c(
      prior(normal(0, 1), class = b),
      prior(normal(0, 2), class = sds),
      prior(normal(0, 1), class = Intercept),
      prior(normal(0, 1), class = sd)
    ),
    logit_w_random_slopes = c(
      prior(normal(0, 1), class = b),
      prior(normal(0, 1), class = sd),
      prior(normal(0, 2), class = sds),
      prior(normal(0, 1), class = Intercept),
      prior(lkj(2), class = cor)
    ),
    logit_phi_w_random_intercepts = c(
      prior(normal(0, 1), class = b),
      prior(normal(0, 2), class = sds),
      prior(normal(0, 1), class = Intercept),
      prior(normal(0, 1), class = sd),
      prior(normal(0, 2), class = b, dpar = phi)
    ),
    logit_phi_w_random_slopes = c(
      prior(normal(0, 1), class = b),
```

```
      prior(normal(0, 2), class = sds),
      prior(normal(0, 1), class = Intercept),
      prior(normal(0, 1), class = sd),
      prior(lkj(2), class = cor),
      prior(normal(0, 2), class = b, dpar = phi)
    )
  )
  l[[set]]
}

has_random_slope_correlation <- function(formula, data) {
  num_cors <- brms::default_prior(formula, data) |>
    filter(group != "") |>
    filter(class == "cor") |>
    nrow()
  num_cors > 0
}

add_loo_criterion <- function(x, ..., use_reloo = FALSE) {
  if (use_reloo) {
    brms::add_criterion(
      x,
      criterion = "loo",
      reloo = TRUE,
      recompile = FALSE,
      ...
    )
  } else {
    brms::add_criterion(
      x,
      criterion = "loo",
      ...
    )
  }
}

brm_args <- function(
    .backend = "cmdstanr",
    .threads = 2,
    .chains = 4,
    .cores = 4,
    .iter = 2000,
    .silent = 0,
    .file_refit = "on_change",
    .refresh = 25,
    ...
) {
  # the .names prevent `file` from partial matching `file_refit`
  defaults <- list(
    backend = .backend,
    threads = .threads,
    chains = .chains,
    cores = .cores,
    iter = .iter,
```

```
    silent = .silent,
    file_refit = .file_refit,
    refresh = .refresh
  )
  dots <- list(...)
  if (is.null(dots$control)) {
    dots$control <- list()
  }
  if (!is.null(dots$adapt_delta)) {
    dots$control$adapt_delta <- dots$adapt_delta
    dots$adapt_delta <- NULL
  }
  if (!is.null(dots$max_treedepth )) {
    dots$control$max_treedepth <- dots$max_treedepth
    dots$max_treedepth <- NULL
  }
  if (length(dots$control) == 0) {
    dots$control <- NULL
  }
  utils::modifyList(defaults, dots)
}
```

References

Bürkner, P.-C. (2017). brms: An R package for Bayesian multilevel models using Stan. *Journal of Statistical Software*, *80*(1), 1–28. https://doi.org/10.18637/jss.v080.i01

Gabry, J., Češnovar, R., & Johnson, A. (2024). *cmdstanr: R interface to CmdStan*. https://mc-stan.org/cmdstanr/

Kay, M. (2024a). ggdist: Visualizations of distributions and uncertainty in the grammar of graphics. *IEEE Transactions on Visualization and Computer Graphics*, *30*(1), 414–424. https://doi.org/10.1109/TVCG.2023.3327195

Kay, M. (2024b). *tidybayes: Tidy data and geoms for Bayesian models*. https://doi.org/10.5281/zenodo.1308151

R Core Team. (2025). *R: A language and environment for statistical computing*. R Foundation for Statistical Computing. https://www.R-project.org/

Revelle, W. (2024). *psych: Procedures for psychological, psychometric, and personality research*. Northwestern University. https://doi.org/10.32614/CRAN.package.psych

Stan Development Team. (2024). *Stan modeling language users guide and reference manual*. https://mc-stan.org

Vehtari, A., Gelman, A., & Gabry, J. (2017). Practical Bayesian model evaluation using leave-one-out cross-validation and WAIC. *Statistics and Computing*, *27*, 1413–1432. https://doi.org/10.1007/s11222-016-9696-4

Wood, S. N. (2017). *Generalized additive models: An introduction with R* (2nd ed.). Chapman; Hall/CRC.
